# Device to Circuit Co‐Design Utilizing High‐Performance PEALD Indium‐Gallium‐Zinc Oxide Thin‐Film Transistor Enabling Technology Node Scaling in Monolithic 3D Systems

**DOI:** 10.1002/advs.202510551

**Published:** 2025-11-16

**Authors:** Wenhui Wang, Tao Zhang, Zelin Yuan, Haoran Peng, Jun Lan, Zhixiong Li, Yongle Wu, Xuewei Feng, Longyang Lin, Feichi Zhou, Panpan Zhang, Yida Li

**Affiliations:** ^1^ School of Microelectronics Southern University of Science and Technology Shenzhen 518055 China; ^2^ School of Integrated Circuits Beijing University of Posts and Telecommunications Beijing 100876 China; ^3^ School of Mechanical Engineering Shanghai Jiao Tong University Shanghai 200240 China; ^4^ State Key Laboratory of Quantum Functional Materials Southern University of Science and Technology Shenzhen 518055 China

**Keywords:** device to circuit co‐design, indium‐gallium‐zinc oxide, oxide circuit, technology computer‐aided design Modeling, technology node scaling, thin‐film transistor

## Abstract

The development of oxide semiconductor devices for monolithic 3D (M3D) integration has largely remained at the device level, limiting progress toward large‐scale applications. To advance this technology, evaluation from device to circuit level—akin to CMOS—is essential. This work presents a comprehensive study of M3D‐compatible technology using indium‐gallium‐zinc oxide (IGZO) thin‐film transistors (TFTs) fabricated via plasma‐enhanced atomic layer deposition (PEALD). The investigation spans material characterization, device performance, technology computer‐aided design (TCAD) Modeling, circuit design, and scaling projections. At device level, comprehensive material and electrical characterizations elucidate intrinsic property interdependencies in IGZO TFTs, which exhibit a field‐effect mobility (*µ_FE_*) up to 116.35 cm^2^ V^−1^·s^−1^, high reliability (△*V_TH_* < 0.15 V), and low variation (△*µ_FE_* < 2%). These results enable accurate TCAD models for system‐level co‐design of unipolar TFT circuits, including pseudo‐CMOS inverters, 5‐stage ring oscillators (ROs), and static random‐access memory (SRAM) cells, all exhibiting robust functionality. For future technology scaling, projections to the 22 nm node show RO frequencies up to 240 MHz and SRAM switching times as low as 0.78 ns, with strong dynamic and noise characteristics. This work offers critical insight into circuit‐level performance of PEALD IGZO TFTs and provides valuable guidance for the implementation of oxide semiconductor‐based M3D systems.

## Graphical abstract

This article presents a holistic investigation of M3D‐compatible oxide semiconductor technology using PEALD‐fabricated IGZO TFTs, spanning device‐level characterization, TCAD Modeling, circuit design, and scaled‐node projections. It demonstrates high field‐effect mobility (up to 116.35 cm^2^ V^−1^·s^−1^), robust reliability (△*V_TH_* < 0.15 V), low variation (△*µ_FE_* < 2%), and forecasts 240 MHz ring oscillators and 0.78 ns SRAM at 22 nm.

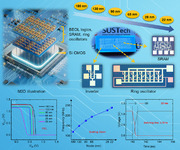

## 1 Introduction

**Figure 1:**
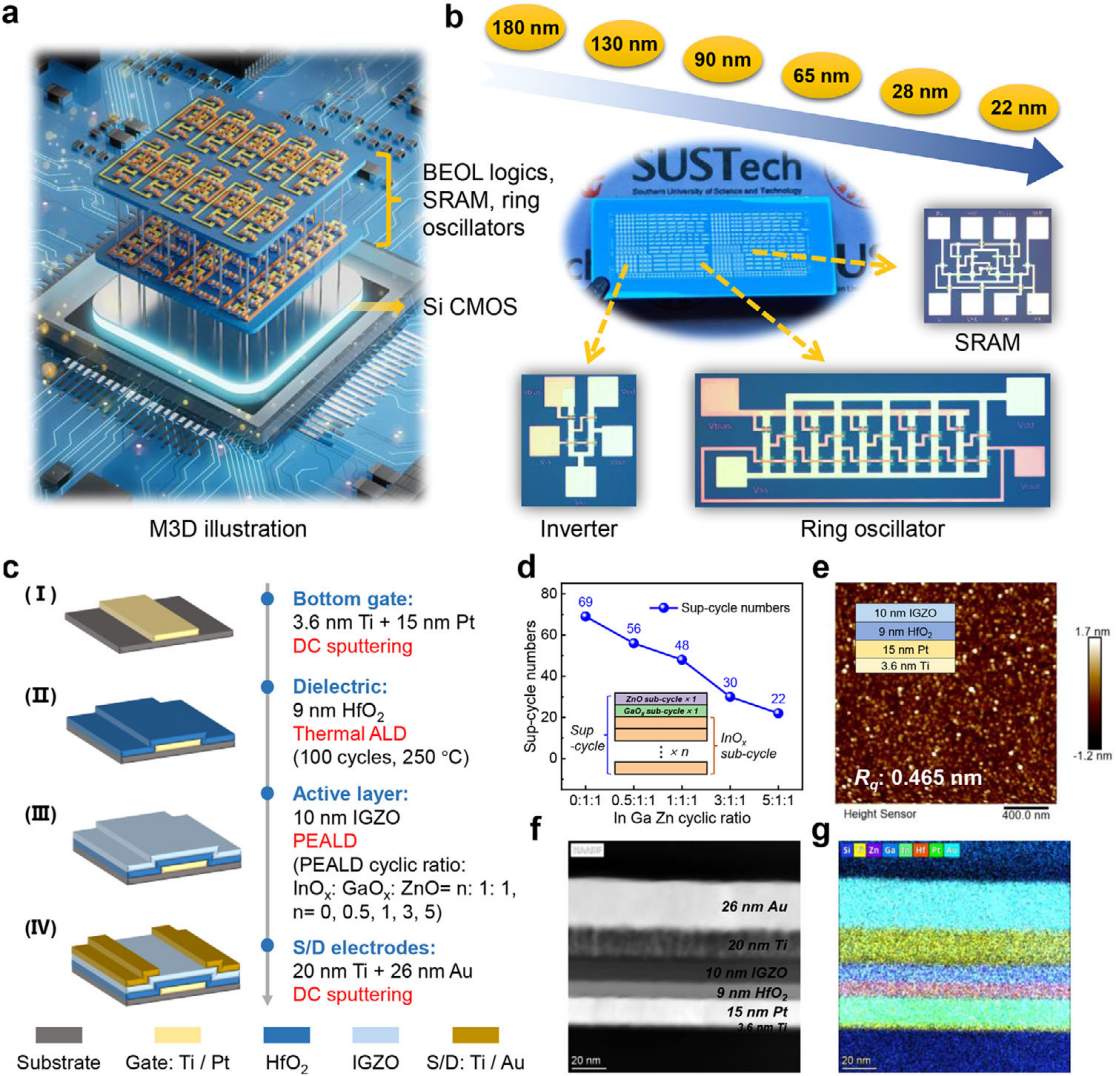
a) The M3D illustration. b) Photo image of the fabricated sample containing the TFT, inverter, ring oscillator, and SRAM. c) Illustration of key fabrication steps of the PEALD IGZO TFTs. d) The super‐cycle numbers for 10 nm‐thick IGZO films with varied InO_x_: GaO_x_: ZnO sub‐cycle ratios, with the diagram of super‐cycle and InO_x_ sub‐cycle in inset. e) The AFM image of the IGZO channel surface. f) The TEM image of the IGZO TFT gate stack, labelled with the different layers. g) The corresponding EDS mapping of elements taken from the gate stack.

In recent years, the demand for computing power has been increasing exponentially as artificial intelligence (AI) and big data analysis applications continue to grow, further exacerbated with the emergence of advanced large language models requiring unprecedented computing power. Moore's Law, which has driven the progress of microelectronics for over fifty years, is going to face significant challenges as transistor scaling in the 2D plane is approaching its physical limit.^[^ ^1^, ^2^ ^]^ 3D computing systems that integrate embedded logic and memory devices monolithically offer a promising solution (**Figure** **1**a), but requires beyond‐silicon (Si) technologies to complement.^[^ ^3^, ^4^, ^5^, ^6^, ^7^ ^]^ Of the various complementary metal‐oxide‐semiconductor (CMOS) back‐end‐of‐line (BEOL) compatible low‐temperature electronic materials such as 2D transition metal chalcogenides (TMDs),^[^ ^5^, ^8^, ^9^, ^10^, ^11^, ^12^ ^]^ carbon nanotubes (CNTs)^[^ ^13^, ^14^, ^15^, ^16^, ^17^ ^]^ and oxide semiconductors,^[^ ^7^, ^18^, ^19^, ^20^, ^21^, ^22^ ^]^ oxide semiconductors are poised to be the immediate candidate for risk production due to its mature process in large‐scale manufacturing using CMOS compatible technology.^[^ ^18^ ^]^

Among potential oxide semiconductors, indium‐gallium‐zinc oxide (IGZO) is undoubtedly the one that stands out the most—not for its superior performance, but for its inherent material stability, which promises reliable performance over the long run.^[^ ^23^, ^24^ ^]^ Nonetheless, the pursuit for the co‐existence of both its performance and stability is still on‐going, driving its integration into circuit‐level implementations within M3D systems. However, the lack in concurrently achieving both acceptable performance and stability, as well as the systematic evaluation of such class of material for circuit‐level implementation suitable for M3D integration at CMOS BEOL, hinders its progress for large‐scale implementation. For instance, at the material and device levels, conventional techniques such as physical vapor deposition (PVD) and chemical vapor deposition (CVD) provide limited control over film quality, making it difficult to precisely modulate defects and stoichiometry for optimal device performance. Furthermore, at the circuit level, current works are far less compared to that on material or device level, with most oxide‐based circuits preliminarily focused on simple demonstrations. System‐level investigations are even scarcer, due to the absence of robust device models capturing adequate device performance variations in a particular technology node.

Hence, in this work, we experimentally addressed the above shortcomings all the way from device to circuit level, and then projected the critical circuit‐level performance to scaled technology nodes to emphasize on the feasibility in advanced integrated circuit (IC) chips (Figure 1b). At the device level, we provided in‐depth insights on plasma‐enhanced atomic layer deposited (PEALD) IGZO thin films in modulating various key materials properties that affect the resulting device performance and stability. The PEALD approach enables deposition at exceptionally low temperatures without further post‐annealing due to the plasma aided effects of precursor reactions, while simultaneously providing precise control over stoichiometry and defect modulation. Thus, an exceptional field‐effect mobility (*µ_FE_*) up to 116.35 cm^2^ V^−1^·s^−1^, outstanding long‐term reliability, and excellent voltage‐bias stability of threshold voltage shift (Δ*V_TH_*) less than 0.15 V were achieved. All the device improvement mechanisms were supported via extensive material characterization—including ultraviolet (UV) absorption, Hall effect measurements, capacitance–voltage (*C–V*) measurements, and X‐ray photoelectron spectroscopy (XPS) analyses. To highlight our process superiority, we characterized more than 100 devices and provided statistical analyses showing the tight distribution of the device performance, followed by the development of a technology computer‐aided design (TCAD) model methodology accounting for the device behaviors in our process technology; the model's performance and robustness were evaluated through a simulation framework that accounts for process variations.

To enable unipolar TFT‐based circuit design, we simulated both pseudo‐enhancement‐load (PEL) and conventional enhancement‐load (EL) inverters using our TCAD device model and experimentally validated them, achieving excellent agreement between simulation and measurement. We then presented 5‐stage ring oscillators (ROs) design framework based on both inverter configurations with functional experimental demonstration. Considering the importance of static random‐access memory (SRAM) process and performance in current CMOS technology, we also demonstrated the design and experimental characterization of a unipolar SRAM based on our IGZO TFT process, exhibiting excellent static and dynamic characteristics, although with the use of n‐only TFTs. This hails a significant milestone as this is the first time that pseudo‐CMOS SRAM based on unipolar oxide semiconductors suitable for BEOL integration has been reported, a key evaluation required to push this technology significantly forward. Finally, to account for their implementation feasibility at advanced technology node, we provided the performance projection of the 5‐stage RO and SRAM cell down to the 22 nm node, with the RO oscillation frequency potentially reaching a high 240 MHz, and SRAM switching time down to a remarkable 0.78 ns with excellent noise margins. Our results, encompassing IGZO TFT performance optimization, device Modeling, circuit design framework, circuit demonstration and technology projection roadmap, are expected to offer valuable guidance for the future integration of IGZO TFT‐based circuitry at CMOS BEOL as Si‐based logic circuits complements.

## 2 Results and Discussion

### 2.1 Fabrication of Devices and Circuits

Key fabrication steps of the staggered, bottom‐gated PEALD IGZO TFTs are shown in Figure 1c. The bottom‐gate electrode of Ti/Pt (3.6/15 nm) was first deposited by DC sputtering. Then, a 9 nm‐thick HfO_2_ used as gate dielectric was deposited via thermal ALD at 250 °C. Following, a 10 nm‐thick IGZO layer was deposited via PEALD at 200 °C using consecutive sequences of IGZO super‐cycles. Each IGZO super‐cycle consisted of a pre‐determined set of InO_x_, GaO_x_, and ZnO sub‐cycle (InO_x_: GaO_x_: ZnO = n: 1: 1, marked as *IGZO_n:1:1_*), with n = 0, 0.5, 1, 3, and 5 to elucidate the effect of indium (In) concentration. IGZO layers of 10 nm thickness were deposited in all splits. Figure 1d shows the super‐cycle numbers for the IGZO films with different InO_x_: GaO_x_: ZnO sub‐cycle ratios, with the diagram of super‐cycle and InO_x_ sub‐cycle in the inset. Finally, the source/drain (S/D) electrodes of Ti/Au (20/26 nm) were deposited using DC sputtering, followed by a lift‐off process. The fabricated TFTs have a channel width (*W_CH_*) of 10 µm and channel length (*L_CH_*) of 5 µm, with optical image shown in Figure S1 (Supporting Information). Details of the fabrication process parameters are provided in the “Experimental Section”.

Figure 1e shows the atomic force microscope (AFM) image of the IGZO channel surface, with a small root mean square roughness (*R_q_*) of 0.465 nm, confirming the high quality of the deposited films. Figure 1f exhibits the transmission electron microscope (TEM) image of the TFT gate stack with clear interfaces between the different layers, with the energy dispersive spectroscopy (EDS) mapping of elements in the gate stack shown in Figure 1g. The grazing incidence X‐ray diffraction (GI‐XRD) patterns exhibit faint and broad peaks at ≈32° with no sharp peaks, confirming the amorphous nature of all the deposited IGZO films,^[^ ^25^, ^26^, ^27^ ^]^ as shown in Figure S2 (Supporting Information).

### 2.2 IGZO TFT Characterizations

**Figure 2:**
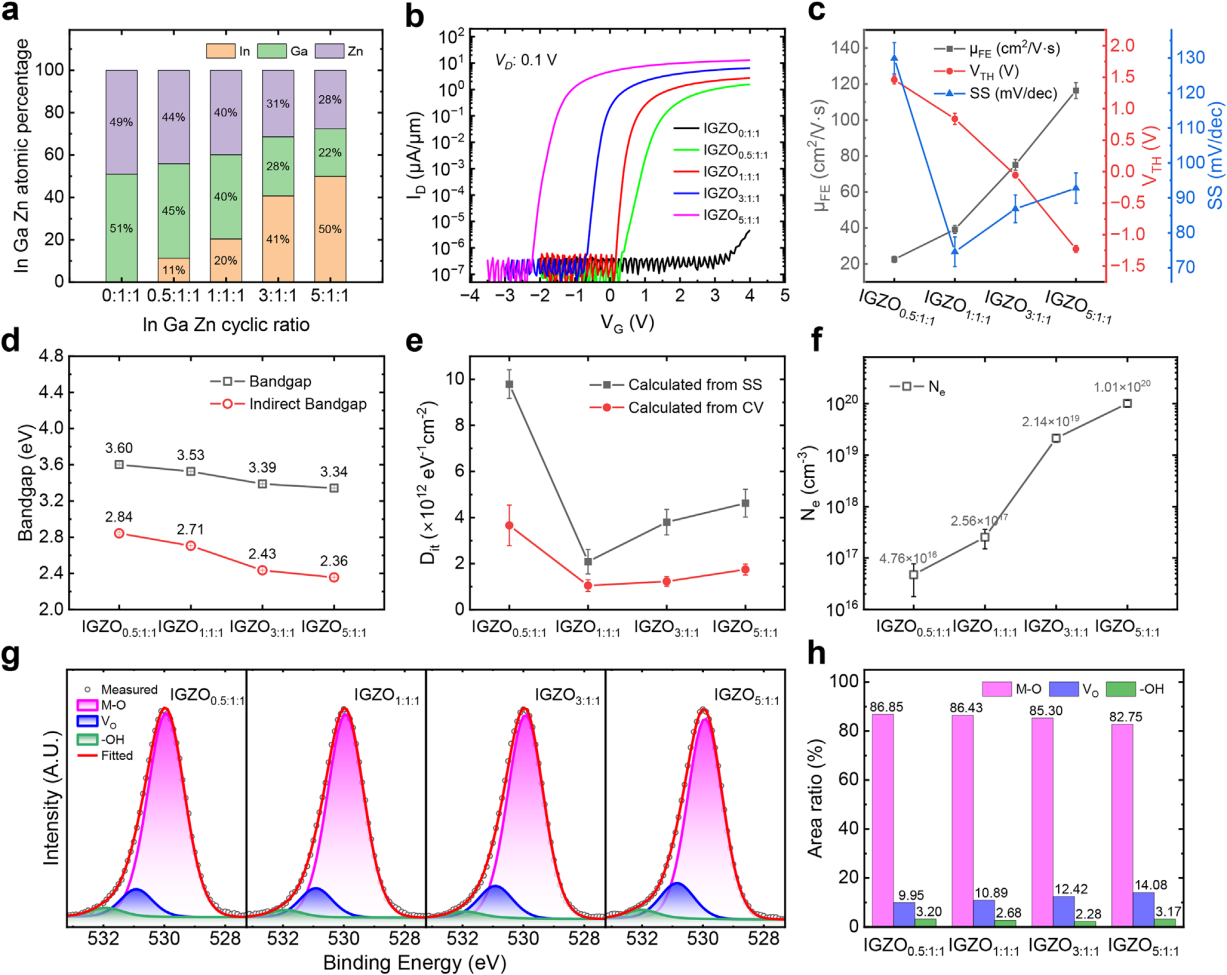
Characterizations and Analyses for IGZO TFTs with different cation ratios. a) The cation atomic percentages in IGZO films with various InO_x_ sub‐cycle numbers in PEALD process. b) The normalized transfer characteristics of the fabricated IGZO TFTs with various In content. c) The extracted *V_TH_*, *µ_FE_*, and *SS* of IGZO TFTs with various In content. d) The bandgap of IGZO films with various In content. e) The *D_it_* in IGZO TFTs with various In content. f) The *N_e_* in IGZO films with various In content. g) The O1s XPS spectra of IGZO films with different InO_x_ sub‐cycle numbers. h) The composition percentages of M‐O, *V_O_*, and ‐OH bonds extracted from O1s XPS spectra.

**Figure** **2**a shows the cation atomic percentages in the prepared IGZO films when the number of InO_x_ sub‐cycles in PEALD process was increased from 0.5 to 5 (corresponding to an increase of In atomic ratio from 11% to 50%), confirmed by XPS analyses. Figure 2b presents the normalized transfer curves (*I_D_*–*V_G_*) of IGZO TFTs with varying In atomic ratios at *V_D_* = 0.1 V. The device was not functional when In was absent in the channel layer. As the In content increased, the TFTs displayed a negative shift in the *V_TH_*. The corresponding key electrical parameters, including *V_TH_*, *µ_FE_*, and subthreshold swing (*SS*), were extracted from the transfer curves of 20 TFTs each, with excellent standard deviation, demonstrating the reproducibility and fabrication reliability for circuit integration (Figure 2c). The detailed extraction methods for the parameters are provided in Note S1 (Supporting Information). When the In atomic ratio in IGZO films was increased from 11% to 50%, the *µ_FE_* improved from 22.54 to 116.35 cm^2^ V^−1^·s^−1^, with a negative *V_TH_* shift from 1.45 to −1.23 V, due to the increased carrier concentration (*N_e_*) in the In‐rich IGZO films. Additionally, the *SS* initially decreased from 130.13 mV dec^−1^ for the *IGZO_0.5:1:1_* TFTs to 74.60 mV dec^−1^ for *IGZO_1:1:1_*, indicating improved switching behavior and reduced trap density. However, as the In content increased further, the *SS* rose to 92.20 mV dec^−1^ for the *IGZO_5:1:1_* sample, suggesting an optimal stoichiometry range beyond which the benefits to switching characteristics are diminished. For comparison, the key performance parameters of TFTs in this work and recent representative ALD‐based IGZO TFTs are summarized in **Table** **1**, demonstrating the best overall performance despite the low thermal budget. The devices with scaled channel thicknesses down to 4 nm were also characterized, as shown in Figure S3 (Supporting Information). The *µ_FE_* rises from 21.89 cm^2^ V^−1^·s^−1^ at *IGZO_1:1:1_* TFT to 65.63 cm^2^ V^−1^·s^−1^ at *IGZO_5:1:1_* TFT while *V_TH_* moves negatively from 1.31 to −0.14 V, mirroring the trend seen in 10 nm‐thick channels. These observations confirm that the underlying mechanism—namely, In‐driven increases in *N_e_* and *V_O_* defects—is intrinsic to the IGZO material itself and does not depend strongly on film thickness.

**Table 1:** Benchmark of reported ALD‐based IGZO TFT performance parameters.

Refs.	Year	Temp. budget [°C]	IGZO thickness [nm]	Dielectric/ thickness [nm]	*I_ON_*/*I_OFF_* ratio	*SS* [mV dec^−1^]	*V_TH_* [V]	*µ_FE_* [cm^2^ V^−1^·s^−1^]
[37]	2017	250	24.3	Al_2_O_3_/100	2 × 10^9^	90	‐	10.4
[38]	2018	180	6	Al_2_O_3_/100	—	120	2.57 ± 0.44	15.1 ± 0.53
[39]	2018	250	13	SiO_2_/100	≈10^8^	300 ± 50	2.41 ± 0.71	22.1 ± 1.3
[35]	2019	450	20	SiO_2_/100	8.9 × 10^8^	260 ± 20	−1.3 ± 0.2	65.5 ± 1.2
[40]	2019	400	16	SiO_2_/100	≈10^7^	410 ± 80	−0.51 ± 0.12	36.6 ± 0.3
[41]	2021	500	13	Al_2_O_3_/4 + HfO_2_/50	3.2 × 10^8^	130 ± 10	0.2 ± 0.24	74 ± 0.91
[42]	2021	350	20	SiO_2_/100	—	360 ± 70	−0.5 ± 0.1	20.1 ± 0.3
[43]	2021	350	20	SiO_2_/100	—	200 ± 10	0.96 ± 0.07	28.17 ± 0.08
[44]	2021	500	20	HfO_2_/33	≈10^4^	540	‐	0.19
[45]	2021	500	5	Al_2_O_3_/10	—	75 ± 1	−0.07 ± 0.08	34.16 ± 1.93
[46]	2021	200	20	Al_2_O_3_/80	—	280	‐	27.6
[47]	2022	400	10	Al_2_O_3_/50	—	140 ± 10	−3.09 ± 0.14	52.48 ± 0.31
[48]	2022	350	20	SiO_2_/100	—	190 ± 10	−1.33 ± 0.16	38.77 ± 1.31
[49]	2022	200	20	Al_2_O_3_/45	—	340	1.5	16.3
[50]	2022	200	20	Al_2_O_3_/80	—	200	−2.51	36.9
[51]	2023	200	20	Al_2_O_3_/40	—	181	—	23.43
[52]	2023	400	10	SiO_2_/0.7 + HfO_2_/4	≈10^8^	64 ± 0.5	−0.12 ± 0.01	22.3 ± 0.5
[53]	2023	250	30	SiO_2_/200	2.2 × 10^8^	200	1.7	8.8
[54]	2023	350	10	Al_2_O_3_/20	—	113.5	−0.47	106.5
[55]	2023	600	8	Al_2_O_3_/13	—	61	0.05	27.6
[56]	2024	400	21	SiO_2_/200	4.78 × 10^6^	210	−1.85	38
[57]	2024	300	18.84	Al_2_O_3_/50	≈10^7^	150	−0.06	26.5
This work	*IGZO_0.5:1:1_*	250	10	HfO_2_/9	≈10^8^	130.13 ± 4.75	1.45 ± 0.06	22.71 ± 1.56
	*IGZO_1:1:1_*	250	10	HfO_2_/9	≈10^8^	74.60 ± 4.27	0.84 ± 0.09	39.17 ± 2.21
	*IGZO_3:1:1_*	250	10	HfO_2_/9	≈10^9^	86.92 ± 3.93	−0.04 ± 0.05	75.39 ± 2.99
	*IGZO_5:1:1_*	250	10	HfO_2_/9	≈10^9^	92.20 ± 4.59	−1.23 ± 0.05	116.05 ± 4.27

To further support the electrical characteristics and parameter variations in IGZO TFTs with varying cation ratios, we performed UV‐vis‐NIR measurements to evaluate the bandgap of IGZO films, *C–V* measurements of metal‐oxide‐semiconductor capacitor (MOSCAP) to analyze the interface state density (*D_it_*) of TFTs, and Hall effect measurements to assess the *N_e_* in IGZO films. Figure 2d presents the bandgap values (including both direct and indirect bandgaps) of the different IGZO films analyzed using UV‐vis‐NIR measurements, with data averaged from five samples each. The bandgap values were calculated through Tauc Plot method,^[^ ^28^ ^]^ with details provided in Note S2 (Supporting Information). IGZO is characterized as an n‐type wide bandgap semiconductor exhibiting both direct and indirect optical transitions. As the In content increased from 11% to 50%, the direct bandgap dropped from 3.60 to 3.34 eV, while the indirect bandgap reduced from 2.84 to 2.36 eV, showing a similar trend for both transitions. The bandgap narrowing can be attributed to the content reduction of Ga_2_O_3_, which inherently has a larger bandgap compared to In_2_O_3_ and ZnO.^[^ ^29^ ^]^ The observed reduction in the bandgap correlates with the negative shift in *V_TH_*, as a narrower bandgap implies that fewer gate‐induced carriers are required to turn on the device. In addition, a smaller indirect bandgap was detected, suggesting the occurrence of indirect band transition caused by the defects formed during the deposition process.^[^ ^30^, ^31^ ^]^ These defects were mainly related to the formation of *V_O_* and interstitial atoms in IGZO films. In addition, the energy difference between the direct and indirect bandgap was calculated to estimate changes in defect transition levels. An increase in energy difference from 0.76 to 0.98 eV indicated a higher concentration of *V_O_*, contributing to the improved *µ_FE_*.

The *SS* of IGZO TFTs is largely determined by trap states at the dielectric‐semiconductor interface, which act as additional capacitances that must be charged before the channel is modulated. The *D_it_* quantifies the density of these trap states per energy interval; a high *D_it_* implies that an applied gate voltage (*V_G_*) initially fills these states rather than shifting the surface potential, thereby degrading *SS*. In this study, the observed trends in the *SS* of the different IGZO TFTs were elucidated using *D_it_* analyses shown in Figure 2e, evaluated separately using two different methods. The one was directly calculated from *SS*, with equation given by:

(1) $$D_{it}=\frac{C_{i}}{q}\left[\frac{SS\cdot log\left(e\right)}{\left(kT/q\right)}-1\right]$$

Where *C_i_* is the unit‐area capacitance of gate dielectric, *k* is the Boltzmann constant, *q* is elementary charge, and *T* is the ambient temperature.^[^ ^32^ ^]^ The other was obtained through high‐ and low‐frequency capacitance measurement (Castagne‐Vapaille method),^[^ ^33^ ^]^ with equation given by:

(2) $$D_{it}=\frac{1}{qA}\left(\frac{C_{ox}C_{lf}}{C_{ox}-C_{lf}}-\frac{C_{ox}C_{hf}}{C_{ox}-C_{hf}}\right)$$

where *C_ox_* is the oxide capacitance, *A* is the area of MOSCAP, *C_lf_* and *C_hf_* are the capacitances of MOSCAPs at low frequency and high frequency, respectively. The *C–V* curves of the IGZO MOSCAPs with various In content are shown in Figure S4 (Supporting Information), where the voltage switching points of the *C–V* curves are consistent with the *V_TH_* of the corresponding TFTs. The energy‐dependent *D_it_* calculated from the *C–V* characteristics of IGZO devices with different In contents shows the similar trend of the *SS* results (Figure S30, Supporting Information). The details of this approach are provided in Note S3 (Supporting Information). The *D_it_* values calculated using both evaluation methods exhibited similar trends (as shown in Figure 2e), confirming the change in *D_it_* of the IGZO TFTs in affecting the *SS* values. The *D_it_* values calculated from *C–V* measurements were lower than that obtained from *SS*, which was because the frequency in the low‐frequency *C–V* measurement, limited by the testing equipment, was not sufficiently low, leading to some interface traps being unable to respond to the AC signal during the measurements. Figure 2f shows the variation trend of *N_e_* measured through a Hall effect system with van der Pauw measurement geometry. As the In content increased from 11% to 50%, the *N_e_* in IGZO films rose by approximately four orders of magnitude, from 4.76 × 10^16^ to 1.01 × 10^20^, largely attributed to the increased unoccupied 5s orbitals with increasing In content, contributing to a lower electron effective mass and enhancing electron mobility.^[^ ^34^, ^35^ ^]^

Further XPS measurements for the different IGZO films are shown in Figure 2g. The O1s peaks were deconvoluted into three distinct sub‐peaks corresponding to the different oxygen species. These included a peak at lower binding energy centered at ≈529.9 eV (magenta curve), corresponding to lattice oxygen or metal‐oxygen (M─O); a peak at medium binding energy centered at ≈530.9 eV (blue curve), associated with *V_O_*; and a peak at higher binding energy centered at ≈531.9 eV (green curve), corresponding to certain impurities such as hydroxyl groups (‐OH).^[^ ^36^ ^]^ The calculated relative area ratios of the M─O, *V_O_*, and ‐OH bonds for different IGZO films are shown in Figure 2h. As the In content in IGZO films increased, the relative proportion of M─O decreased while the *V_O_* increased, with no significant variation in the ‐OH. This behavior stems from In's larger ionic radius and weaker In‐O bond energy, which destabilize the oxide network and promote *V_O_* formation. In oxide semiconductors, *V_O_* is generally regarded as the source of shallow donor states that contribute to charge carrier generation. Consequently, the increase of *V_O_* content directly results in a higher *N_e_* and improved mobility. In summary, all the evidence points to the increase of In content within the IGZO films leading to a higher density of *V_O_*, thereby elevating *N_e_* and enhancing *µ_FE_*, highlighting the critical role of compositional tuning in optimizing the electrical properties of IGZO TFTs.

The long‐term reliability, voltage‐bias, and temperature‐bias stability of the optimized IGZO TFTs were also assessed. As depicted in Figure S5 (Supporting Information), the devices exhibited negligible change after 120 days in a humid‐air environment despite the absence of channel encapsulation. Under positive bias temperature stress (PBTS), they displayed a negligible positive *V_TH_* shift (Δ*V_TH_*) of 0.07 ± 0.03 V, and under negative bias temperature stress (NBTS), a small negative Δ*V_TH_* of −0.15 ± 0.05 V was observed over 3600 s (Figure S6, Supporting Information). Under temperature bias, the devices exhibited modest Δ*V_TH_* ≈ −0.14 V at 80 °C and ‐0.65 V at 120 °C, and these shifts are fully reversible upon cooling back to room temperature (Figure S7, Supporting Information). Hence, we demonstrated that with proper material properties modulation and deposition technique, both performance and reliability over multiple devices can be achieved concurrently for circuits implementation presented in the following sections. In addition, the advantages of PEALD IGZO deposition process compared with sputter process is supplemented in Note S4 (Supporting Information). The influence of PEALD process parameters on IGZO TFT properties were also systematically investigated, and supplemented in Note S5 (Supporting Information). The contact resistance (*R_C_*) was also evaluated via the transfer length method (TLM) to assess its impact on mobility, as detailed in Note S6 (Supporting Information).

### 2.3 IGZO TFT Modeling

**Figure 3:**
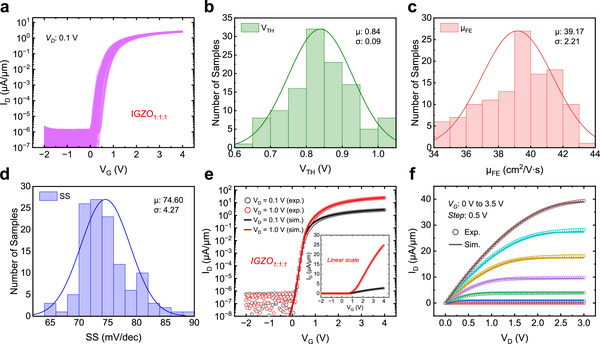
IGZO TFT Modeling. a) The normalized transfer curves (*I_D_*–*V_G_*) of 120 fabricated IGZO TFTs. b–d) The statistical histograms of the extracted *V_TH_*, *µ_FE_*, and *SS* from the120 devices, with the Gaussian fit curves. e,f) Experimental (symbols) and simulated (solid lines) transfer and output characteristics of the IGZO TFT, with an inset in e) showing linear scale plots.

To extend the applications of IGZO TFTs to the logic circuit level, a TCAD device model using Sentaurus was developed to accurately capture the unique DC characteristics of IGZO TFTs. In this work, the *IGZO_1:1:1_* TFT was chosen for our device Modeling and circuit‐level simulations because it offers a well‐balanced performance, that is, a modest mobility (39.17 cm^2^ V^−1^·s^−1^) together with a positive *V_TH_* (0.84 V) and small *SS* (74.60 mV dec^−1^). While mobility is an important performance metric, a positive *V_TH_* is especially important to eliminate high off‐state leakage and standby power. Considering the reasonably good mobility and desired positive *V_TH_*, our choice of *IGZO_1:1:1_* provides an optimal balance for low‐power digital design, ensuring both noise margin and leakage control. In addition, the strategies to mitigate the *V_TH_* shift in high‐mobility compositions is discussed in Note S7 (Supporting Information). **Figure** **3**a exhibits the normalized transfer curves (*I_D_*–*V_G_*) of 120 fabricated *IGZO_1:1:1_* TFTs at *V_D_* = 0.1 V. The *I_D_*–*V_G_* clusters show good uniformity and small *V_TH_* variation. Figure 3b–d shows the statistical histograms of the extracted parameters of *V_TH_*, *µ_FE_*, and *SS* from all the devices, exhibiting gaussian distributions with the mean value (*µ*) and standard deviation (σ) labelled. These electrical parameters served as the foundation for device Modeling, with details provided in the “Experimental Section”.

Figure 3e,f displays the experimental (symbols) and simulated (solid lines) transfer and output characteristics of the IGZO TFTs. The excellent agreement between the experimental and simulated results were achieved after the extracted *V_TH_*, *µ_FE_*, *SS*, physical and geometrical parameters were fed into the model. The experimental and simulated families of *I_D_*–*V_G_* curves (*V_D_*: 0.1 to 1 V, *step*: 0.1 V) for the *IGZO_1:1:1_* TFT are shown in Figure S8 (Supporting Information), showing both logarithmic‐scale and linear‐scale plots to clearly exhibit the fitness of curves in subthreshold and saturation regions.

### 2.4 Simulation and Experimental Demonstration for IGZO TFT‐Based Inverter

**Figure 4:**
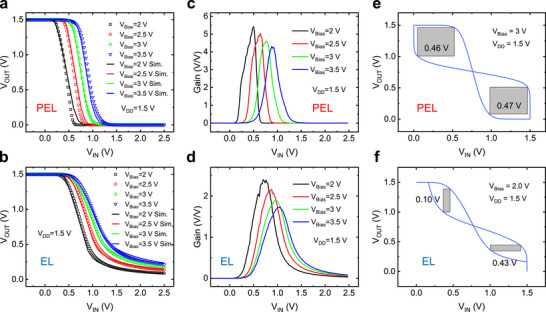
Simulation and experimental demonstration of IGZO TFT‐based inverter. a,b) The simulated and experimentally measured VTCs of the PEL and EL inverters at *V_DD_* of 1.5 V and *V_Bias_* varying from 2 to 3.5 V in voltage step of 0.5 V. c,d) The corresponding voltage gains of the PEL and EL inverters, extracted from VTCs. e,f) The corresponding noise margin (NM) of the PEL and EL inverters, extracted from VTCs.

Inverter serves as the fundamental building block in digital logic circuits. Utilizing the calibrated TFT model, two inverter configurations—namely, PEL and EL—were designed and systematically evaluated. The circuit diagrams and corresponding optical micrographs of both inverters are presented in Figure S9 (Supporting Information). The voltage transfer curves (VTCs) were optimized through simulation by adjusting the TFT parameters (i.e., *W_CH_* and *L_CH_*) to achieve the best voltage gain and noise margin (NM) for each configuration, with the optimized dimensions summarized in Table S1 (Supporting Information). **Figure** **4**a,b compares the simulated and experimentally measured VTCs of the PEL and EL inverters, showing excellent agreement. Both simulations and experiments were performed at *V_DD_* = 1.5 V, while *V_Bias_* was varied from 2 to 3.5 V in step of 0.5 V. Under a constant *V_DD_*, the VTCs exhibited a positive shift as *V_Bias_* increased, primarily due to the increasing *V_IM_*, which reduced the impedance of transistor *T_3_* (see Figure S9, Supporting Information). The EL inverters functioned, but did not achieve rail‐to‐rail operation due to the finite load impedance of the pull‐up transistor (*T_1_*). In contrast, the PEL inverters achieved rail‐to‐rail operation, switching *V_OUT_* from *V_DD_* to near 0 V (*V_SS_*) owing to the inclusion of a second stage that formed a pseudo‐CMOS structure, thereby increasing the overall load impedance of the pull‐up transistor.

Figure 4c,f presents the voltage gain and NM (both derived from the VTCs) for the PEL and EL inverters. The gains of both inverters decreased as *V_Bias_* increased from 2 to 3.5 V, attributed to the reduced impedance of the pull‐up transistor (*T_1_*) with increasing *V_Bias_*. The PEL inverters achieved a low noise margin (*NM_L_*) of 0.46 V and high noise margin (*NM_H_*) of 0.47 V at *V_DD_* = 1.5 V and *V_Bias_* = 3 V, while the EL inverters achieved a *NM_L_* of 0.10 V and *NM_H_* of 0.43 V at *V_DD_* = 1.5 V and *V_Bias_* = 2 V. The PEL inverters exhibited a larger gain and NM compared to EL inverters, primarily attributed to the pseudo‐CMOS structure at the cost of doubling the number of TFTs. Additional VTCs and gains for both inverters under varying *V_DD_* are shown in Figures S10 and S11 (Supporting Information). A benchmark table (Table S2, Supporting Information) summarizes key parameters of the two inverters, confirming that the PEL configuration offers superior robustness due to its two‐stage pseudo‐CMOS structure.

### 2.5 Experimental Demonstration and Projection for IGZO TFT‐Based Ring Oscillator

**Figure 5:**
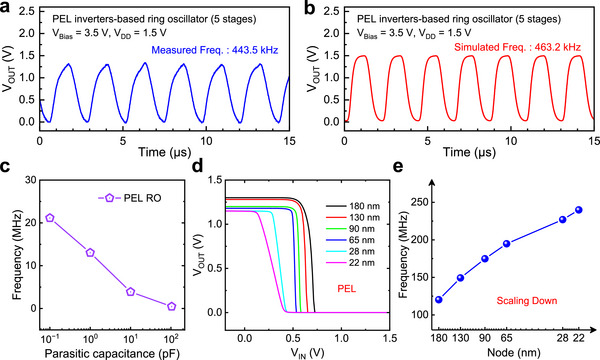
Experimental demonstration and projection for IGZO TFT‐based ring oscillator. a,b) The experimentally measured and simulated output waveforms of the PEL inverter‐based 5‐stage RO, respectively, showing good agreement. c) Projected oscillation frequency as a function of parasitic capacitance in the PEL inverter‐based RO. d) Projected VTCs of inverters across various technology nodes. e Projected oscillation frequency for ROs across various technology nodes.

To explore the merits and potential of high‐performance IGZO TFTs at the circuit level, we designed and experimentally demonstrated 5‐stage ROs based on both PEL and EL inverters. The circuit diagram and optical micrographs of the fabricated ROs are presented in Figure S12 (Supporting Information). **Figure** **5**a,b shows the experimentally measured and simulated output waveforms of the PEL inverter‐based 5‐stage RO at *V_DD_* = 1.5 V and *V_Bias_* = 3.5 V, respectively. The measured oscillation frequency of the PEL inverter‐based RO was 443.5 kHz, whereas the simulated frequency was 463.2 kHz. By contrast, the measured oscillation frequency of the EL inverter‐based RO was 324.9 kHz, and the simulated value was 323.9 kHz (as shown in Figure S13, Supporting Information). Although the PEL inverter‐based RO required twice as many TFTs and therefore incurred a larger area penalty, they achieved a higher oscillation frequency compared to the EL inverter‐based RO, which can be attributed to the higher voltage gain of the PEL inverters. To validate the robustness of the simulation results, 1000 Monte Carlo simulations were performed for the PEL inverter‐based RO, with the amplitude and frequency distributions shown in Figure S14 (Supporting Information). The measured oscillation frequencies closely matched the corresponding simulation values for both two types of ROs, when considering the parasitic capacitances existed in the fabricated ROs. However, in practical integrated circuit design and manufacturing, such parasitic capacitances are substantially reduced—commonly below 0.1 pF—by minimizing unnecessary wiring and contact pads. To evaluate the true performance of the proposed RO, the oscillation frequency as a function of parasitic capacitance was projected; the oscillation frequency of the IGZO TFT‐based RO can reach ≈21.1 MHz when the parasitic capacitance is projected to 0.1 pF (Figure 5c).

To further evaluate the scalability of the proposed ROs at more advanced technology nodes, we performed projections based on the calibrated TCAD device model. The projected VTCs of inverters at various technology nodes are shown in Figure 5d, while Figure 5e plots the corresponding predicted frequencies of RO (the corresponding output waveforms shown in Figure S15, Supporting Information). As technology nodes advance, the achievable frequency increases markedly, surpassing 240 MHz at the 22 nm node. These findings demonstrated that the operating frequency of IGZO TFT‐based RO can be substantially improved through both parasitic‐capacitance reduction and technology scaling, underscoring the promise of IGZO TFTs for high‐speed electronic applications. The performance metrics for recently reported oxide‐based ROs are summarized in Table S3 (Supporting Information). Our proposed ROs achieved largest voltage swing (88%) and oscillation frequency (443.5 kHz) at lowest *V_DD_* (1.5 V), which can be attributed to the excellent performance of the optimized IGZO TFTs.

### 2.6 Experimental Demonstration and Simulation for IGZO TFT‐Based SRAM

To further explore the potential of high‐performance IGZO TFTs at the logic circuit level, we designed and experimentally demonstrated a SRAM cell based on PEL inverters, a critical module in all CMOS technology nodes. The typical 6‐T SRAM architecture was employed, comprising two cross‐coupled PEL inverters and two access transistors (*T_C1_*, *T_C2_*), as depicted in Figure S16 (Supporting Information). The device dimensions, particularly the *W_CH_* and *L_CH_* of the transistors within SRAM cell, are critical parameters that influence both the read/write operations and overall stability. Specifically, a higher drivability of the pull‐down transistors (*T_A2_*, *T_B2_*) relative to the access transistors is necessary for robust read operations, whereas a lower drivability of the pull‐up transistors (*T_A1_*, *T_B1_*) compared to the access transistors is preferred for efficient write operations.^[^ ^58^ ^]^ These channel dimensions were optimized through simulations in Sentaurus, and the resulting optimized TFT sizes are summarized in Table S4 (Supporting Information).

**Figure 6:**
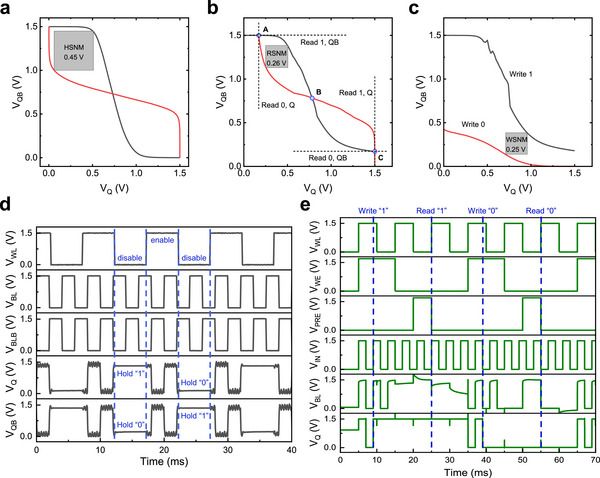
Static and dynamic characteristics of IGZO TFT‐based SRAM. a–c) The VTCs and extracted static noise margin (SNM) during hold, read, and write operations. d) Dynamic response of the SRAM during continuous write and hold operations. e) Simulated dynamic characteristic during continuous write and read operations.

The static characteristics of the SRAM cell—including the hold static noise margin (HSNM), read static noise margin (RSNM), and write static noise margin (WSNM)—serve as critical indicators of its stability and sensitivity. A high HSNM value of 0.45 V, which corresponds to 60% when normalized by *V_DD_*/2, was achieved, indicating excellent stability of the SRAM cell in its data hold state (**Figure** **6**a). A RSNM value of 0.26 V (34.67%, normalized by *V_DD_*/2) was achieved, confirming that the cell can reliably output one‐bit binary data during read operations (Figure 6b). A WSNM value of 0.25 V (33.33%, normalized by *V_DD_*/2) was realized, demonstrating that one‐bit binary data can be stably written into the cell (Figure 6c). Additionally, the N‐curve method was also employed to further evaluate the read/write performance (Figure S17, Supporting Information). The detailed evaluation methods and analyses for HSNM, RSNM, WSNM and N‐curve, are supplemented in Note S8 (Supporting Information). Although only n‐type TFTs were used in the SRAM, all the static characteristics, including HSNM, RSNM, and WSNM, exhibited large values and good robustness, which can be attributed to the high performance of optimized IGZO TFTs and pseudo‐CMOS structure of inverters. To further evaluate the static characteristics of the proposed SRAM at advanced technology nodes (extending down to a 22 nm dimension), we conducted simulations using Sentaurus to assess the HSNM, RSNM and WSNM (Figures S18–S20, Supporting Information). The static characteristics exhibited optimal performance at the 65 nm node, but deteriorated with further scaling (Figure S21, Supporting Information). This behavior originated from pronounced short‐channel effects in sub‐65 nm devices which degraded the *SS* and induced *V_TH_* drift, consequently flattening the slope of the inverter's VTC and reducing the SRAM noise margin (Figure S22, Supporting Information).

The dynamic characteristics were also evaluated. Figure 6d illustrates the dynamic response during write and hold operations at a bit line (*BL*) frequency of 250 Hz. In this test configuration, when the word line (*WL*) was enabled (the *WL* voltage was set to *V_DD_*), the cell could update the stored data at *Q* and *QB* nodes reliably. Conversely, when the *WL* was disabled (the *WL* voltage was set to *GND*), the cell effectively retained the stored logic “0” or “1,” demonstrating robust data hold capability. During write operations, the switching time measured was 97 µs (Figure S23, Supporting Information), whereas simulations projected a reduction to as low as 0.78 ns at the 22 nm node (Figure S24, Supporting Information). Due to measurement limitations, the dynamic response of the read operation was characterized through simulation using Sentaurus. To enable sequential write and read operations, the SRAM cell requires peripheral circuits, specifically the pre‐charge and write‐driver modules. As illustrated in Figure S25 (Supporting Information), the write‐enable signal (*WE*) controls the write operation, while the pre‐charge signal (*PRE*) determines the execution of the pre‐charge process. To compensate for threshold voltage loss, the voltages of both *PRE* and *WE* were elevated to *V_DD_* + *V_TH_*. Figure 6e exhibits the simulated dynamic response during continuous write and read operations, and the simulated timing sequence is described in details in Note S9 (Supporting Information). The designed SRAM cell exhibited excellent performance in both static and dynamic evaluations. Table S5 (Supporting Information) presents the performance comparison between our proposed design and several state‐of‐the‐art SRAMs, demonstrating that our approach achieved optimal overall performance at the lowest *V_DD_*, despite with a unipolar TFT design. In addition, the limitations in scaling IGZO TFTs for dense logic circuits are discussed in Note S10 (Supporting Information), while the discussion of the M3D integration are expanded in Note S11 (Supporting Information).

## 3 Conclusion

In summary, we provided a comprehensive assessment of PEALD IGZO TFTs, as well as its derived functional circuitries suitable for M3D integration. At the device level, an exceptional *µ_FE_* up to 116.35 cm^2^ V^−1^·s^−1^, outstanding long‐term reliability, and excellent voltage‐bias stability of Δ*V_TH_* less than 0.15 V were achieved. All the device improvement mechanisms were supported via extensive material characterization—including UV absorption, Hall effect measurements, *C–V* measurements, and XPS analyses. To enable unipolar TFT‐based circuit design, a TCAD model for the optimized IGZO TFT was established via Sentaurus. Both PEL and EL inverters were simulated and experimentally validated using the established device model, achieving excellent agreement between simulation and measurement. We then presented 5‐stage ROs design framework based on both inverter configurations with functional experimental demonstration. We also demonstrated the design and experimental characterization of a unipolar SRAM based on PEL inverters for the first time, exhibiting excellent static and dynamic characteristics, although with the use of n‐only TFTs. Finally, to account for their implementation feasibility at advanced technology node, we provided the performance projection of the 5‐stage RO and SRAM cell down to the 22 nm node, with the RO oscillation frequency potentially reaching a high 240 MHz, and SRAM switching time down to a remarkable 0.78 ns with excellent noise margins. Our results, encompassing IGZO TFT performance optimization, device Modeling, circuit design framework, circuit demonstration and technology projection roadmap, are expected to offer valuable guidance for the future integration of IGZO TFT‐based circuitry at CMOS BEOL.

## 4 Experimental Section

### Device Fabrication

The IGZO TFTs and circuits were fabricated on the SiO_2_/Si substrate. The fabrication process started with the standard cleaning steps with the SC‐1 and SC‐2, followed by ultrasonic cleaning using acetone and isopropyl alcohol (IPA). The bottom‐gate electrode, composed of Ti and Pt with thickness of 3.6 and 15 nm respectively (as confirmed by TEM imaging), was first deposited using a DC sputtering system (PVD75, Kurt J. Lesker) onto a p^+^‐doped Si substrate coated with 285 nm‐thick thermally oxidized SiO_2_ layer. The Argon (Ar) was used as carrier gas, and the working pressure was set as 3 mTorr (corresponding the Ar flow of 12.4 sccm). The sputtering powers of Ti and Pt were set as 120 and 100 W, respectively. The film growth rates of Ti and Pt were 0.56 and 1.38 Å s^−1^, respectively. Then, a 9 nm‐thick HfO_2_ layer was deposited as the gate dielectric by thermal atomic layer deposition (ALD) over 100 cycles, using tetrakis (dimethylamino) hafnium (TDMAH) as the Hf precursor and H_2_O as the reactant respectively at 250 °C. In the ALD process for HfO_2_, the pulse time and purge time for TDMAH were set to 0.1 and 20 s, while those for H_2_O were 0.06 and 40 s, respectively. The carrier gas (99.999% N_2_) flow rates for TDMAH and H_2_O were set as 200 sccm. The TDMAH source bottle was maintained at 65 °C, while H_2_O source was kept at room temperature during the deposition. Next, the 10 nm‐thick IGZO active channel layer was deposited using PEALD at 200 °C. In the PEALD process, the (3‐dimethylamino‐propyl) dimethyl indium (DADI), trimethyl gallium (TMGa), and diethyl zinc (DEZ) were used as In, Ga and Zn precursors respectively, while oxygen plasma was used as reactant. The DADI source bottle was heated to 80 °C, while TMGa and DEZ source bottles were kept at room temperature during the process. The pulse time and purge time for DADI, TMGa and DEZ were set to 0.05 and 40 s, respectively. The carrier gas (99.999% N_2_) flow rates for DADI, TMGa and DEZ were set to 200 sccm. The O_2_ flow rate was set to 200 sccm, while the plasma power was set as 500 W. The stay time and purge time for oxygen plasma were set to 20 and 15 s, respectively. Both the HfO_2_ gate dielectric and IGZO channel regions were defined via standard lithography followed by a buffered oxide etch (BOE) etching process. The BOE etching rates for HfO_2_ and IGZO layers were ≈0.1 and 1 nm s^−1^ through calibration, respectively. After that, the source/drain electrodes (comprising 20 nm Ti and 26 nm Au) were deposited using DC sputtering followed by a lift‐off process (same as the bottom gate). The sputtering power of Au was set as 50 W, while the film growth rate was 1 Å s^−1^. Finally, pad opening for devices and circuits was conducted through standard lithography followed by BOE etching.

The fabrication process for the proposed ROs and SRAM cell closely followed that of IGZO TFTs, with one additional process step: the incorporation of an additional inter‐layer deposition of a 75 nm‐thick SiO_2_ layer, deposited using RF sputtering, between the overlapping bottom and top metal interconnects. This additional process step is critical for ensuring the proper insulation and performance of the integrated device structure.

### Characterizations and Measurements

All film thicknesses and the refractive indices of IGZO films were measured using an ellipsometer (TF‐UVISEL). The channel surface roughness was measured using AFM (Dimension Edge). Cross‐sectional observation of TFT gate stack was performed with TEM (Talos F200X G2), operating at 120 kV. The cross‐sectional specimens of IGZO TFT stack for TEM measurement were prepared via focused ion beam (FIB) (Helios 5 CX). The crystallinity of IGZO films was analyzed using GI‐XRD (Rigaku Smartlab) under parallel beam (PB) optical path mode, in which the grazing incidence was set as 0.5° while the voltage and current for X‐ray were 40 kV and 150 mA, respectively. The chemical bonding and elemental compositions were analyzed using XPS (ESCALAB 250Xi), with monochromatic Al Kα X‐ray source. The absorption spectra of IGZO films (deposited on quartz substrate) were measured using UV‐vis‐NIR Spectrometer (PerkinElmer Lambda 950).

The carrier concentrations of IGZO films were measured using a Hall effect system (Nanometrics HL5500PC), with van der Pauw measurement geometry. Current–voltage (*I–V*) characteristics of IGZO TFTs, capacitance–voltage (*C–V*) curves of Metal‐HfO_2_‐IGZO‐Metal MOSCAP structure, VTCs of all inverters and SNMs of SRAM cells were measured with a semiconductor parameter analyzer (Keithley 4200‐SCS) at room temperature under ambient conditions. The output waveforms of ROs and dynamic responses of SRAM cells were measured using an oscilloscope (Tektronix MSO64B). The rectangular waves for write operation of SRAM were generated by a signal generator (Tektronix AFG1022).

### TCAD Modeling

All simulations were performed using Sentaurus. IGZO was modelled as a bulk semiconductor and channel transport approximated via the drift‐diffusion method. Band tail states were incorporated to accurately represent the doping conditions in IGZO. The onset of the doping‐dependent mobility feature was used to account for mobility degradation resulting from carrier scattering by charged impurity ions within IGZO. Additionally, the carrier velocity saturation phenomenon in short‐channel devices under high electric fields was captured using the Canali model^[^ ^59^ ^]^ based on the Caughey‐Thomas formulation.^[^ ^60^ ^]^ Material parameters were iteratively tailored to fit the measured transfer and output characteristics until the on‐state performance error was reduced to less than 1%, and the results presented in Table S6 (Supporting Information). The details of the TCAD device model calibration are supplemented in Note S12 (Supporting Information).

### Circuits Simulation

With the calibrated TCAD model for IGZO TFTs, the impact of device scalability on circuit‐level performance was systematically investigated by shrinking the channel length from 5 µm to 22 nm. Benchmark circuits, including a 5‐stage RO and a 6T SRAM, were simulated using mixed‐mode in Sentaurus Device. For the 5‐stage RO, oscillation frequencies were extracted from transient simulation results as a function of technology node, with a load capacitor introduced at each stage to model the lumped parasitic effects of interconnects. To accommodate scaling‐induced changes, the load capacitor values were dynamically adjusted for each technology node; the initial parasitic capacitor value for the 5 µm node was determined through calibration against experimental data, and the corresponding supply voltages and load capacitor values for each node are presented in Table S7 (Supporting Information). For the SRAM circuit, both transient and quasistationary simulations were performed. The dynamic read and write operations were analyzed via transient simulation, while the hold, read, and write SNM were evaluated through quasistationary analysis.

## Conflict of Interest

The authors declare no conflict of interest.

## Supporting information



Supporting Information

## Data Availability

The data that support the findings of this study are available from the corresponding author upon reasonable request.

## References

[1] M. S. Lundstrom , M. A. Alam , Science 2022, 378, 6621. 10.1126/science.ade2191 36395227

[2] F. Zhu , P. Xu , J. Zong , Appl. Comput. Eng. 2023, 10, 307.

[3] Y. Cheng , X. Guo , V. F. Pavlidis , Integration 2022, 85, 97.

[4] M. Xie , Y. Jia , C. Nie , Z. Liu , A. Tang , S. Fan , X. Liang , L. Jiang , Z. He , R. Yang , Nat. Commun. 2023, 14, 5952. 37741834 10.1038/s41467-023-41736-2 PMC10517937

[5] J. H. Kang , H. Shin , K. S. Kim , M. K. Song , D. Lee , Y. Meng , C. Choi , J. M. Suh , B. J. Kim , H. Kim , A. T. Hoang , B. I. Park , G. Zhou , S. Sundaram , P. Vuong , J. Shin , J. Choe , Z. Xu , R. Younas , J. S. Kim , S. Han , S. Lee , S. O. Kim , B. Kang , S. Seo , H. Ahn , S. Seo , K. Reidy , E. Park , S. Mun , et al., Nat. Mater. 2023, 22, 1470. 38012388 10.1038/s41563-023-01704-z

[6] D. Jayachandran , N. U. Sakib , S. Das , Nat. Rev. Electr. Eng. 2024, 1, 300.

[7] S. Yuvaraja , H. Faber , M. Kumar , N. Xiao , G. I. Maciel García , X. Tang , T. D. Anthopoulos , X. Li , Nat. Electron. 2024, 7, 768.

[8] B. Radisavljevic , A. Radenovic , J. Brivio , V. Giacometti , A. Kis , Nat. Nanotechnol. 2011, 6, 147. 21278752 10.1038/nnano.2010.279

[9] K. Kang , S. Xie , L. Huang , Y. Han , P. Y. Huang , K. F. Mak , C. J. Kim , D. Muller , J. Park , Nature 2015, 520, 656. 25925478 10.1038/nature14417

[10] M. Sivan , Y. Li , H. Veluri , Y. Zhao , B. Tang , X. Wang , E. Zamburg , J. F. Leong , J. X. Niu , U. Chand , A. V. Thean , Nat. Commun. 2019, 10, 5201. 31729375 10.1038/s41467-019-13176-4 PMC6858359

[11] B. Tang , H. Veluri , Y. Li , Z. G. Yu , M. Waqar , J. F. Leong , M. Sivan , E. Zamburg , Y. W. Zhang , J. Wang , A. V. Thean , Nat. Commun. 2022, 13, 3037. 35650181 10.1038/s41467-022-30519-w PMC9160094

[12] B. Tang , M. Sivan , J. F. Leong , Z. Xu , Y. Zhang , J. Li , R. Wan , Q. Wan , E. Zamburg , A. V. Y. Thean , npj 2D Mater. Appl. 2024, 8, 74.

[13] A. D. Franklin , M. C. Hersam , H.‐S. P. Wong , Science 2022, 378, 726. 36395207 10.1126/science.abp8278

[14] C. Liu , Y. Cao , B. Wang , Z. Zhang , Y. Lin , L. Xu , Y. Yang , C. Jin , L. M. Peng , Z. Zhang , ACS Nano 2022, 16, 21482. 36416375 10.1021/acsnano.2c10007

[15] C. Fan , X. Cheng , Y. Xie , F. Liu , X. Deng , M. Zhu , Y. Gao , M. Xiao , Z. Zhang , ACS Nano 2023, 17, 10987. 37256833 10.1021/acsnano.3c03190

[16] K. Wang , X. Liu , Z. Zhao , L. Li , J. Tong , Q. Shang , Y. Liu , Z. Zhang , Carbon 2023, 205, 540.

[17] Y. Lin , Y. Cao , S. Ding , P. Zhang , L. Xu , C. Liu , Q. Hu , C. Jin , L.‐M. Peng , Z. Zhang , Nat. Electron. 2023, 6, 506.

[18] T. Kim , C. H. Choi , J. S. Hur , D. Ha , B. J. Kuh , Y. Kim , M. H. Cho , S. Kim , J. K. Jeong , Adv. Mater. 2023, 35, 2204663. 10.1002/adma.202204663 35862931

[19] M. Si , Z. Lin , Z. Chen , X. Sun , H. Wang , P. D. Ye , Nat. Electron. 2022, 5, 164.

[20] J. Zhang , W. Wang , J. Zhu , J. Wang , C. Zhao , T. Zhu , Q. Ren , Q. Liu , R. Qiu , M. Zhang , X. Wang , H. Meng , K. C. Chang , S. Zhang , M. Chan , Adv. Funct. Mater. 2023, 33.

[21] Z. H. Li , T. C. Chiang , P. Y. Kuo , C. H. Tu , Y. Kuo , P. T. Liu , Adv. Sci. (Weinh) 2023, 10, 2205481. 36658711 10.1002/advs.202205481 PMC10037976

[22] J. Lu , M. Shen , X. Feng , T. Tan , H. Guo , L. Lin , F. Zhou , Y. Li , Nano Lett. 2024, 24, 15260. 39561989 10.1021/acs.nanolett.4c03742

[23] J. Troughton , D. Atkinson , J. Mater. Chem. C 2019, 7, 12388.

[24] S. Y. Lee , Trans. Electr. Electron. Mater. 2020, 21, 235.

[25] K. Nomura , H. Ohta , A. Takagi , T. Kamiya , M. Hirano , H. Hosono , Nature 2004, 432, 488. 15565150 10.1038/nature03090

[26] D. M. Lynch , B. Zhu , B. D. A. Levin , D. A. Muller , D. G. Ast , R. G. Greene , M. O. Thompson , Appl. Phys. Lett. 2014, 105, 262103.

[27] J. S. Lim , F. K. Yam , J. Mater. Sci.: Mater. Electron. 2023, 34.

[28] J. Tauc , R. Grigorovici , A. Vancu , Phys. Status Solidi 1966, 15, 627.

[29] T. Ando , A. B. Fowler , F. Stern , Rev. Mod. Phys. 1982, 54, 437.

[30] H. J. Quah , Z. Hassan , W. F. Lim , J. Alloys Compd. 2019, 777, 736.

[31] N. Kasim , Z. Hassan , W. F. Lim , H. J. Quah , J. Mater. Sci.: Mater. Electron. 2020, 31, 9705.

[32] C. Liu , Y. Sun , H. Qin , Y. Liu , S. Wei , Y. Zhao , IEEE Electron Device Lett. 2019, 40, 415.

[33] R. Engel‐Herbert , Y. Hwang , S. Stemmer , J. Appl. Phys. 2010, 108, 124101.

[34] A. Olziersky , P. Barquinha , A. Vilà , C. Magaña , E. Fortunato , J. R. Morante , R. Martins , Mater. Chem. Phys. 2011, 131, 512.

[35] J. Sheng , T. Hong , H. M. Lee , K. Kim , M. Sasase , J. Kim , H. Hosono , J. S. Park , ACS Appl. Mater. Interfaces 2019, 11, 40300. 31584254 10.1021/acsami.9b14310

[36] J. Lan , Z. Li , Z. Chen , Q. Zhu , W. Wang , M. Zaheer , J. Lu , J. Liang , M. Shen , P. Chen , K. Chen , G. Zhang , Z. Wang , F. Zhou , L. Lin , Y. Li , Adv. Electron. Mater. 2023, 9, 2201250.

[37] S. M. Yoon , N. J. Seong , K. Choi , G. H. Seo , W. C. Shin , ACS Appl. Mater. Interfaces 2017, 9, 22676. 28653825 10.1021/acsami.7b04637

[38] S. J. Yoon , N. J. Seong , K. Choi , W. C. Shin , S. M. Yoon , RSC Adv. 2018, 8, 25014. 35542140 10.1039/c8ra03639j PMC9082295

[39] M. H. Cho , H. Seol , H. Yang , P. S. Yun , J. U. Bae , K.‐S. Park , J. K. Jeong , IEEE Electron Device Lett. 2018, 39, 688.

[40] M. H. Cho , H. Seol , A. Song , S. Choi , Y. Song , P. S. Yun , K.‐B. Chung , J. U. Bae , K.‐S. Park , J. K. Jeong , IEEE Trans. Electron Devices 2019, 66, 1783.

[41] M. H. Cho , C. H. Choi , H. J. Seul , H. C. Cho , J. K. Jeong , ACS Appl. Mater. Interfaces 2021, 13, 16628. 33793185 10.1021/acsami.0c22677

[42] D.‐G. Kim , S.‐H. Ryu , H.‐J. Jeong , J.‐S. Park , ACS Appl. Electron. Mater. 2021, 3, 3530.

[43] W.‐H. Choi , K. Kim , S.‐G. Jeong , J.‐H. Han , J. Jang , J. Noh , K.‐S. Park , J.‐J. Kim , S.‐Y. Yoon , W. Jeon , J.‐S. Park , IEEE Trans. Electron Devices 2021, 68, 6147.

[44] H. J. Ryoo , N. J. Seong , K. J. Choi , S. M. Yoon , Nanotechnology 2021, 32, 255201. 10.1088/1361-6528/abcbc4 33207327

[45] S.‐G. Jeong , H.‐J. Jeong , J.‐S. Park , IEEE Trans. Electron Devices 2021, 68, 1670.

[46] S.‐H. Moon , S.‐H. Bae , Y. H. Kwon , N.‐J. Seong , J.‐H. Yang , Y.‐H. Kim , K.‐J. Choi , C.‐S. Hwang , S.‐M. Yoon , ACS Appl. Electron. Mater. 2021, 3, 4849.

[47] K. S. Yoo , D.‐G. Kim , S. Lee , W.‐B. Lee , J.‐S. Park , Ceram. Int. 2022, 48, 18803.

[48] Y. S. Kim , W. B. Lee , H. J. Oh , T. Hong , J. S. Park , Adv. Mater. Interfaces 2022, 9, 2200501.

[49] S. H. Bae , J. H. Yang , Y. H. Kim , Y. H. Kwon , N. J. Seong , K. J. Choi , C. S. Hwang , S. M. Yoon , ACS Appl. Mater. Interfaces 2022, 14, 31010. 35785988 10.1021/acsami.2c07258

[50] D.‐H. Lee , Y.‐H. Kwon , N.‐J. Seong , K.‐J. Choi , G. Kim , S.‐M. Yoon , ACS Appl. Electron. Mater. 2022, 4, 6215.

[51] S.‐H. Moon , Y.‐H. Kwon , N.‐J. Seong , K.‐J. Choi , S.‐M. Yoon , IEEE Electron Device Lett. 2023, 44, 1128.

[52] C. H. Choi , T. Kim , M. J. Kim , S. H. Yoon , J. K. Jeong , IEEE Trans. Electron Devices 2023, 70, 2317.

[53] Q.‐Z. Chen , C.‐Y. Shi , M.‐J. Zhao , P. Gao , W.‐Y. Wu , D.‐S. Wuu , R.‐H. Horng , S.‐Y. Lien , W.‐Z. Zhu , IEEE Electron Device Lett. 2023, 44, 448.

[54] D. G. Kim , H. Choi , Y. S. Kim , D. H. Lee , H. J. Oh , J. H. Lee , J. Kim , S. Lee , B. Kuh , T. Kim , H. Y. Kim , J. S. Park , ACS Appl. Mater. Interfaces 2023, 15, 31652. 37350067 10.1021/acsami.3c05678

[55] D. G. Kim , W. B. Lee , S. Lee , J. Koh , B. Kuh , J. S. Park , ACS Appl. Mater. Interfaces 2023, 15, 36550. 37489641 10.1021/acsami.3c06517

[56] P. Wen , C. Peng , Z. Chen , X. Ding , F.‐H. Chen , G. Yan , L. Xu , D. Wang , X. Sun , L. Chen , J. Li , X. Li , J. Zhang , Appl. Phys. Lett. 2024, 124, 133501.

[57] Z. Chen , J. Yang , X. Ding , X. Li , J. Zhang , IEEE Trans. Electron Devices 2024, 71, 1963.

[58] J. Zhang , W. Wang , J. Zhu , C. Wang , T. Zhu , C. Zhao , J. Wang , S. Zhang , X. Wang , K. C. Chang , H. Meng , M. Chan , M. Zhang , ACS Nano 2024, 18, 3362. 38227541 10.1021/acsnano.3c10182

[59] C. Canali , G. Majni , R. Minder , G. Ottaviani , IEEE Trans. Electron Devices 1975, 22, 1045.

[60] D. M. Caughey , R. E. Thomas , Proc. IEEE 1967, 55, 2192.

