# Supplementary material for: Device to Circuit Co‐Design Utilizing High‐Performance PEALD Indium‐Gallium‐Zinc Oxide Thin‐Film Transistor Enabling Technology Node Scaling in Monolithic 3D Systems
Source: Adv Sci (Weinh). 2025 Nov 16;13(6):e10551. doi: 10.1002/advs.202510551 (PMC12866809; doi:10.1002/advs.202510551)
Supplement: Supplementary file 1 — Supporting Information [file ADVS-13-e10551-s001.pdf]

Supporting Information

Device to Circuit Co-Design Utilizing High-Performance PEALD Indium-Gallium-Zinc Oxide Thin-Film Transistor Enabling Technology Node Scaling in Monolithic Three-Dimensional Systems

Wenhui Wang, Tao Zhang, Zelin Yuan, Haoran Peng, Jun Lan, Zhixiong Li, Yongle Wu, Xuewei Feng, Longyang Lin, Feichi Zhou, Panpan Zhang*, and Yida Li**

W. Wang, Z. Yuan, H. Peng, J. Lan, Z. Li, L. Lin, F. Zhou, Y. Li
School of Microelectronics, Southern University of Science and Technology, Shenzhen 518055, China
E-mail: liyd3@sustech.edu.cn, zhoufc@sustech.edu.cn

T. Zhang, Y. Wu, P. Zhang
School of Integrated Circuits, Beijing University of Posts and Telecommunications, Beijing 100876, China
E-mail: tanji_ic@bupt.edu.cn

X. Feng
School of Mechanical Engineering, Shanghai Jiao Tong University, Shanghai, 200240, China

Supporting Figures

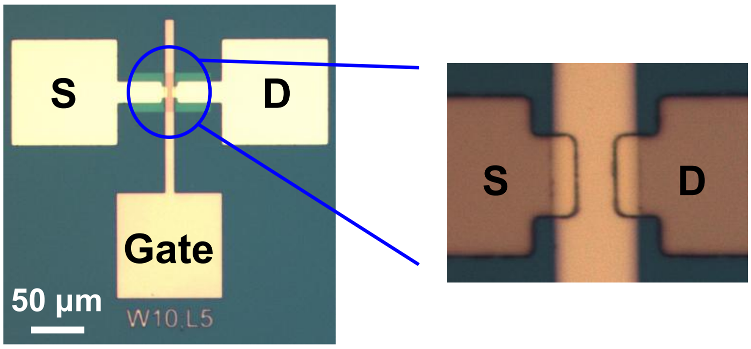

Figure S1. The optical micrograph of fabricated TFT and zoom-in channel region.

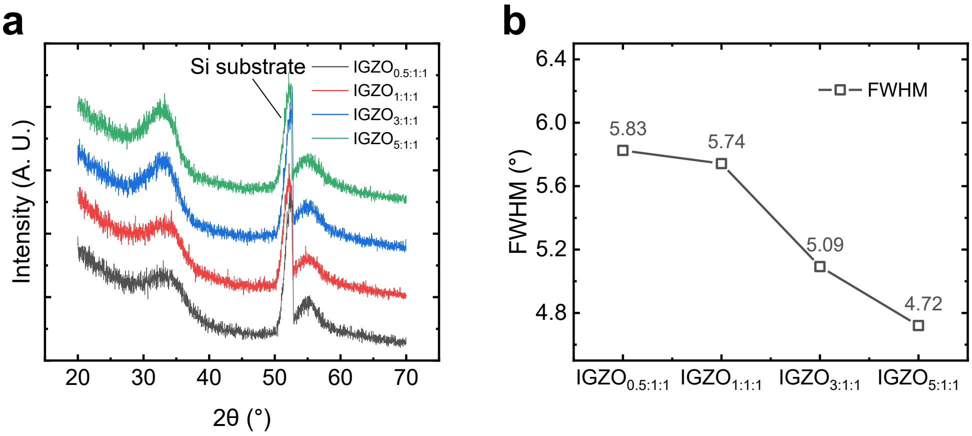

Figure S2. a The GI-XRD patterns of IGZO films with different InO_x sub-cycle numbers in PEALD process. The faint and broad peaks may result from the small In_2O_3 crystallites in the IGZO films, as In_2O_3 typically exhibits polycrystalline properties. **b** The corresponding full width at half maximum (FWHM) of GI-XRD patterns. As the In concentration in the IGZO films increases, the FWHM decreases, indicating an increase in crystallite size.

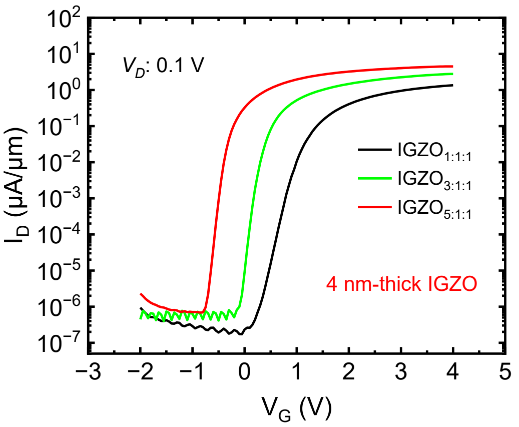

Figure S3. The transfer characteristics of the IGZO TFTs with varying In content, with scaled channel thicknesses of 4 nm.

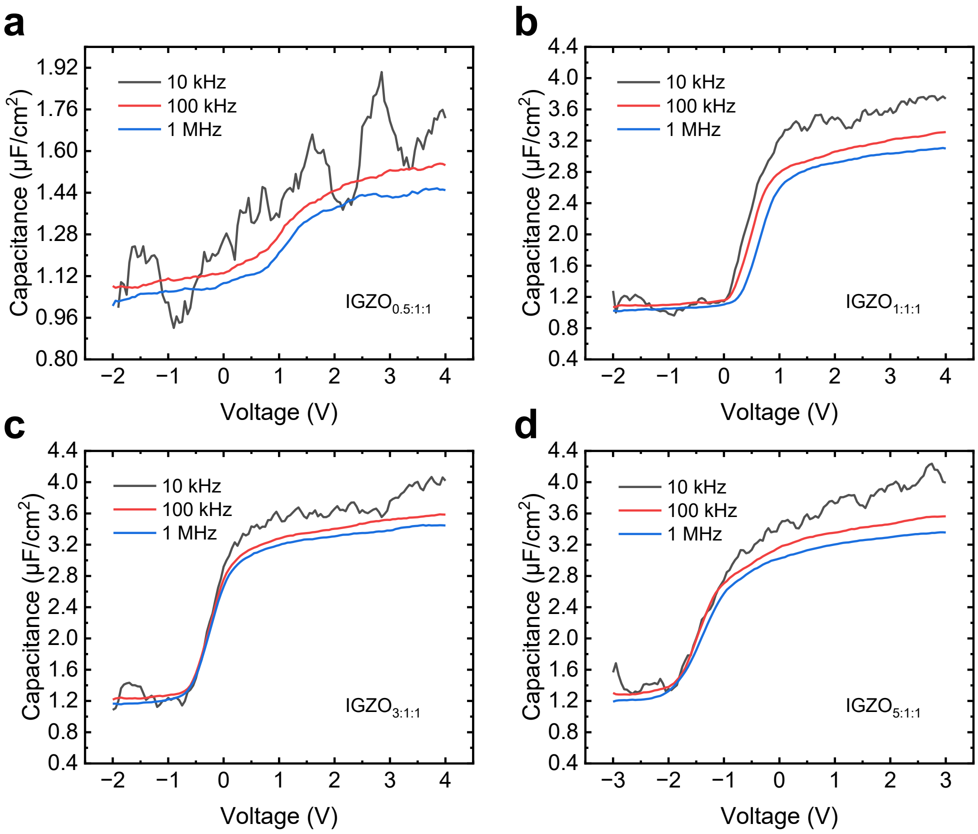

Figure S4. The C-V curves of **a** *IGZO*$_{0.5:1:1}$ MOSCAP, **b** *IGZO*$_{1:1:1}$ MOSCAP, **c** *IGZO*$_{3:1:1}$ MOSCAP, and **d** *IGZO*$_{5:1:1}$ MOSCAP under different measurement frequency.

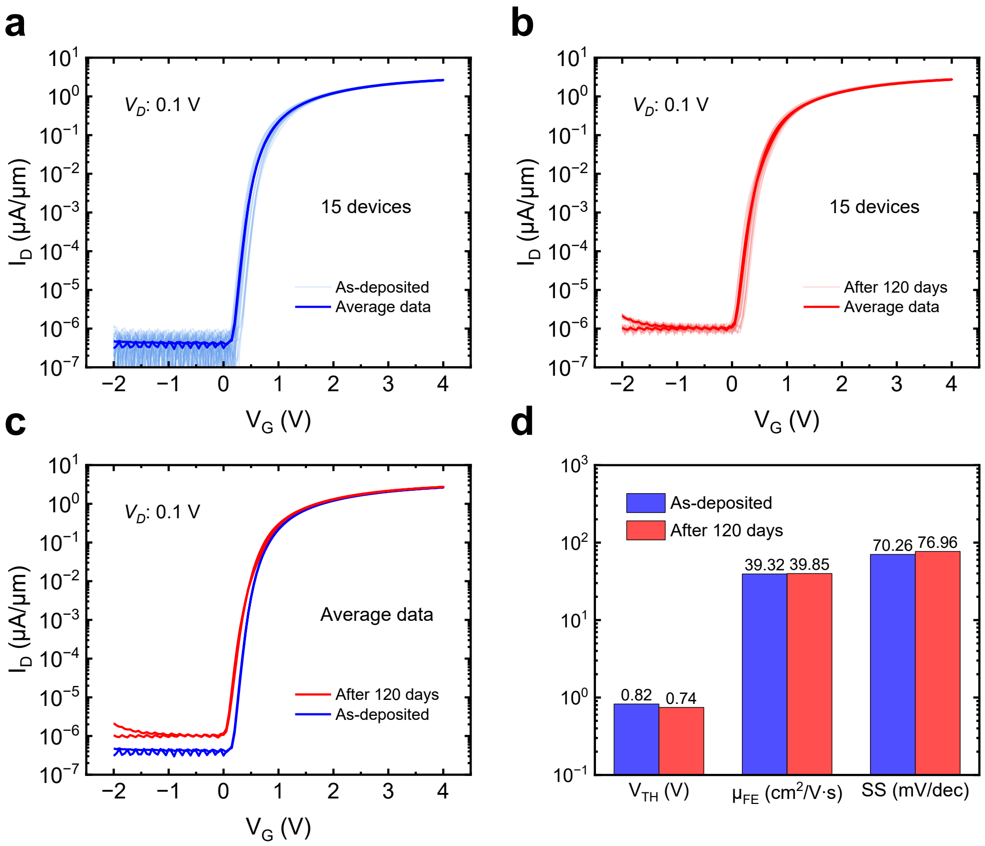

Figure S5. The long-term stability characterization of the optimized IGZO TFTs. **a** The transfer curves of as-deposited devices. **b** The transfer curves of devices measured after 120 days in a humid-air environment. **c** The average transfer curves of devices under both conditions from **a** and **b**. **d** The extracted electrical parameters for long-term stability comparison.

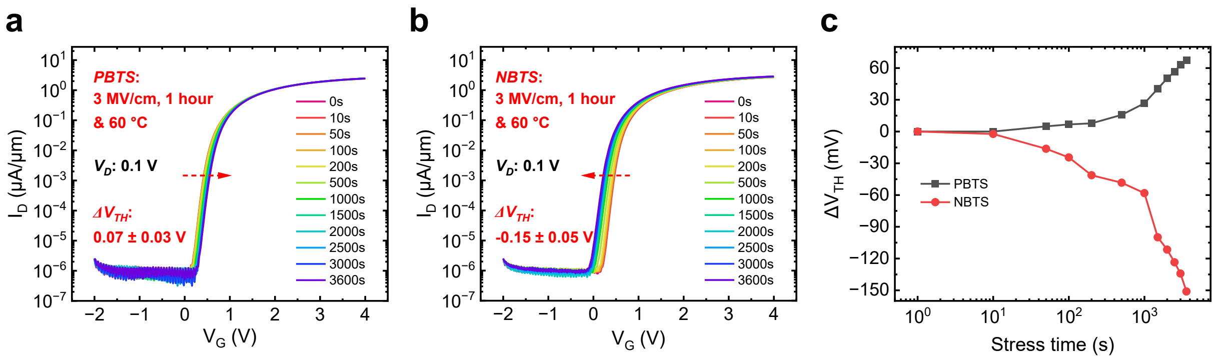

Figure S6. The voltage-bias stability characterization of the optimized IGZO TFTs. **a** The positive bias temperature stress (PBTS) and **b** negative bias temperature stress (NBTS) characterizations at gate bias electric field of ±3 MV/cm and 60 °C for 3600 s, respectively. **c** The ΔV_{TH} as a function of stress time.

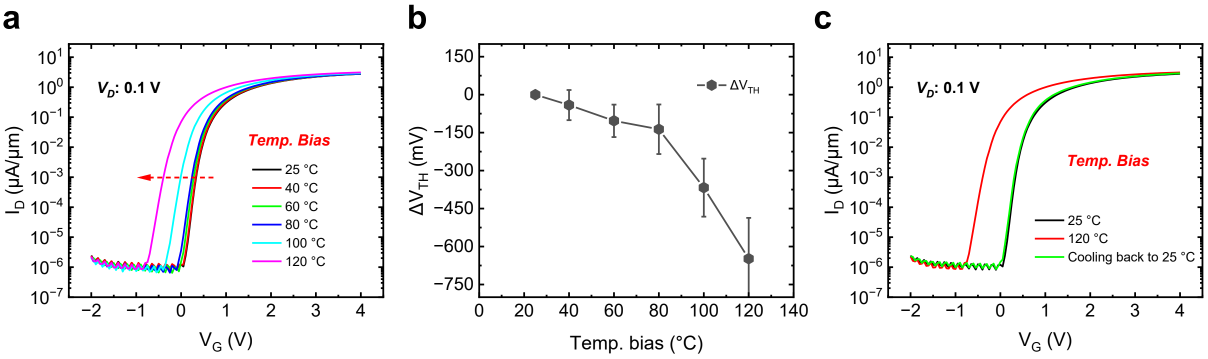

Figure S7. The temperature-bias stability of the optimized IGZO TFTs. **a** Transfer characteristic measured at V_D = 0.1 V under temperature-bias conditions from 25 to 120 °C; the dashed arrow marks the direction of the V_{TH} shift. **b** Extracted V_{TH} shift (ΔV_{TH}) as a function of temperature bias. **c** Transfer characteristics measured at 25 °C, 120 °C and upon cooling back to 25 °C.

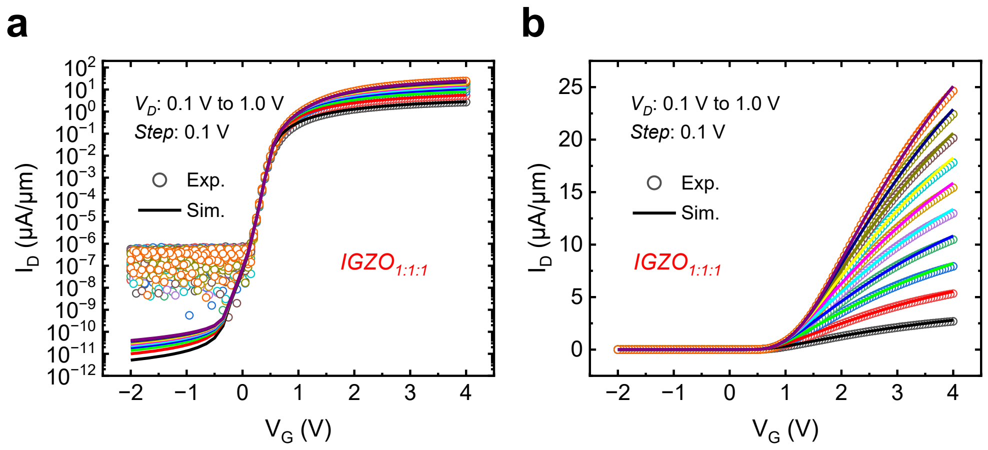

Figure S8. The experimental and simulated family of I_D-V_G curves. The transfer curves measured at V_D from 0.1 V to 1 V in step of 0.1 V, showing both logarithmic-scale and linear-scale plots to clearly exhibit the fitness of curves in subthreshold and saturation regions.

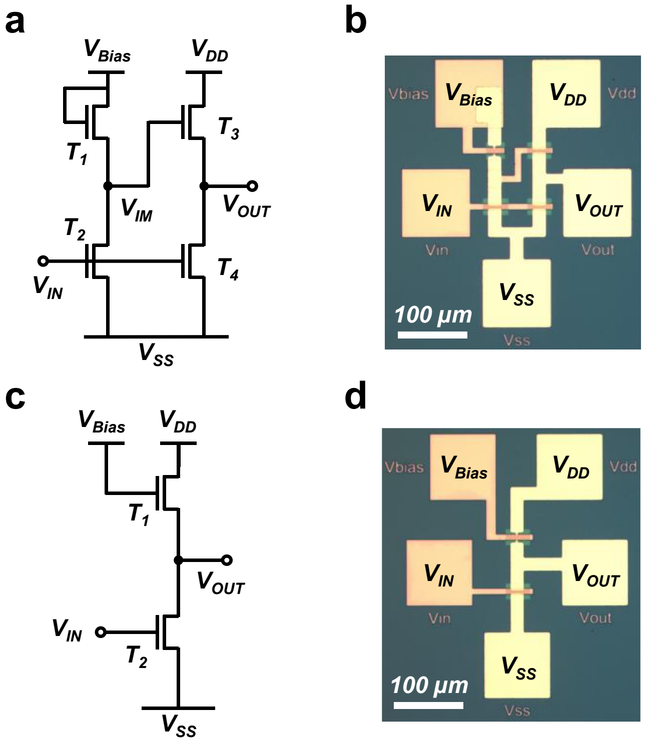

Figure S9. a, **b** The circuit diagram and optical micrograph of the PEL inverter. **c**, **d** The circuit diagram and optical micrograph of the EL inverter.

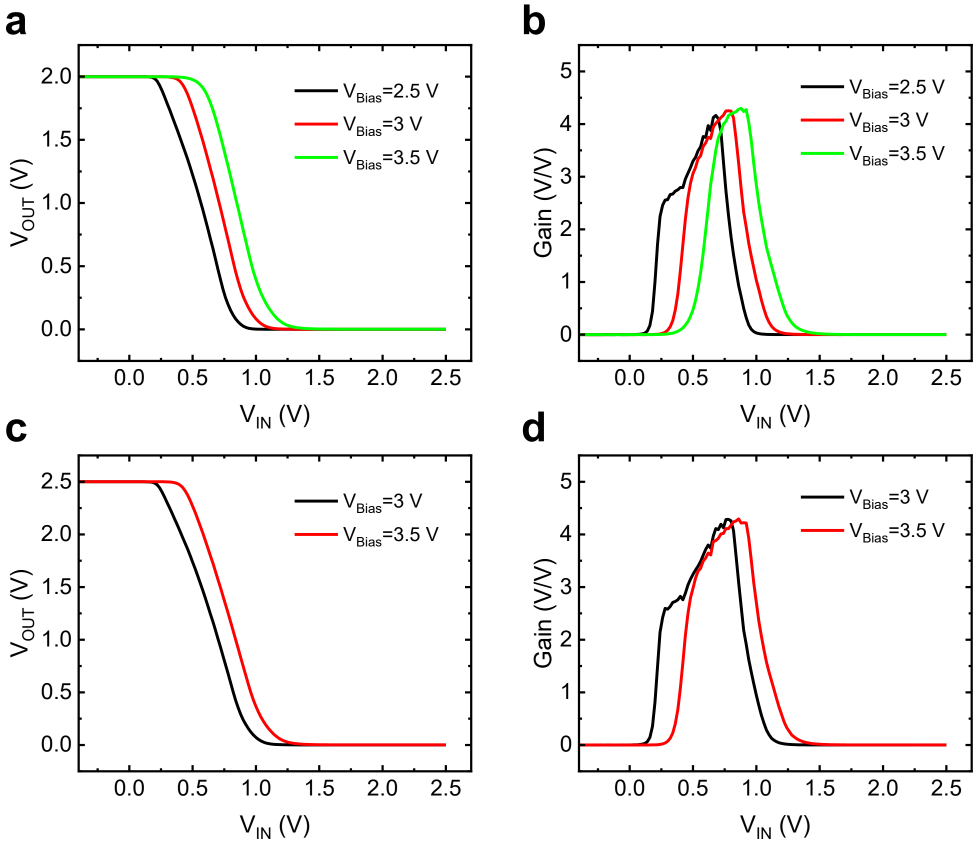

Figure S10. PEL inverter VTCs and gains. **a** The experimental measured VTCs and **b** the corresponding voltage gains of the PEL inverter at V_{DD} of 2 V and V_{Bias} varying from 2.5 V to 3.5 V in voltage step of 0.5 V. **c** The experimental measured VTCs and **d** the corresponding voltage gains of the PEL inverter at V_{DD} of 2.5 V and V_{Bias} varying from 3 V to 3.5 V in voltage step of 0.5 V.

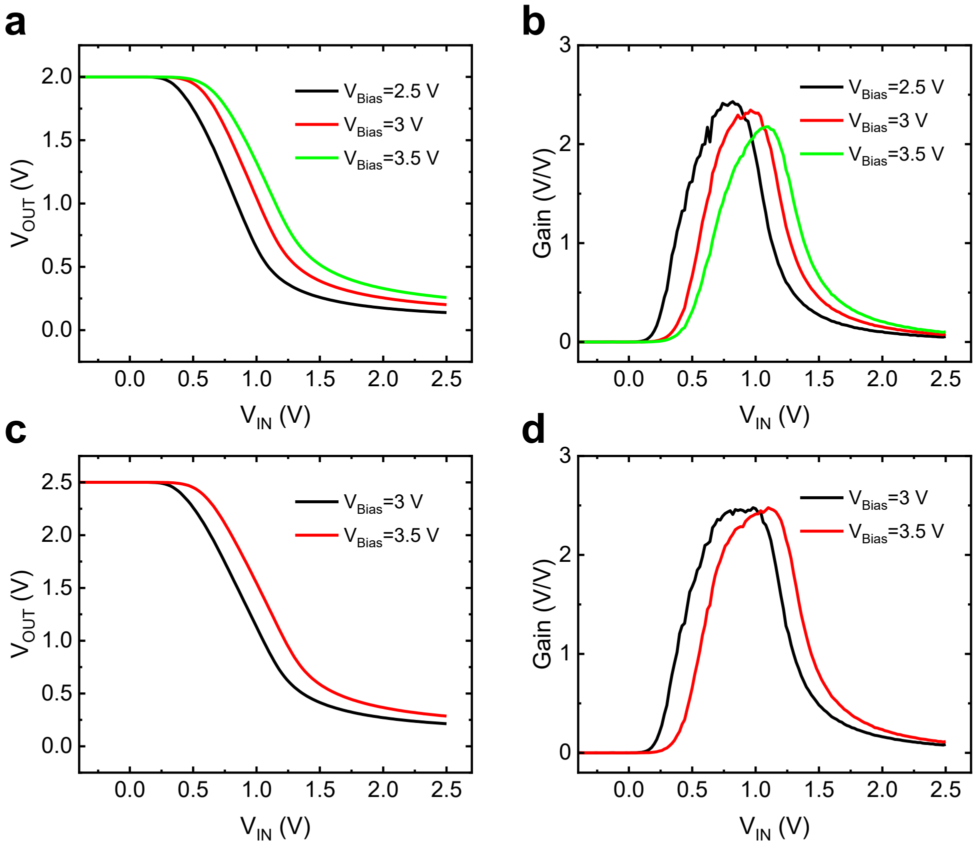

Figure S11. EL inverter VTCs and gains. **a** The experimental measured VTCs and **b** the corresponding voltage gains of the EL inverter at V_{DD} of 2 V and V_{Bias} varying from 2.5 V to 3.5 V in voltage step of 0.5 V. **c** The experimental measured VTCs and **d** the corresponding voltage gains of the EL inverter at V_{DD} of 2.5 V and V_{Bias} varying from 3 V to 3.5 V in voltage step of 0.5 V.

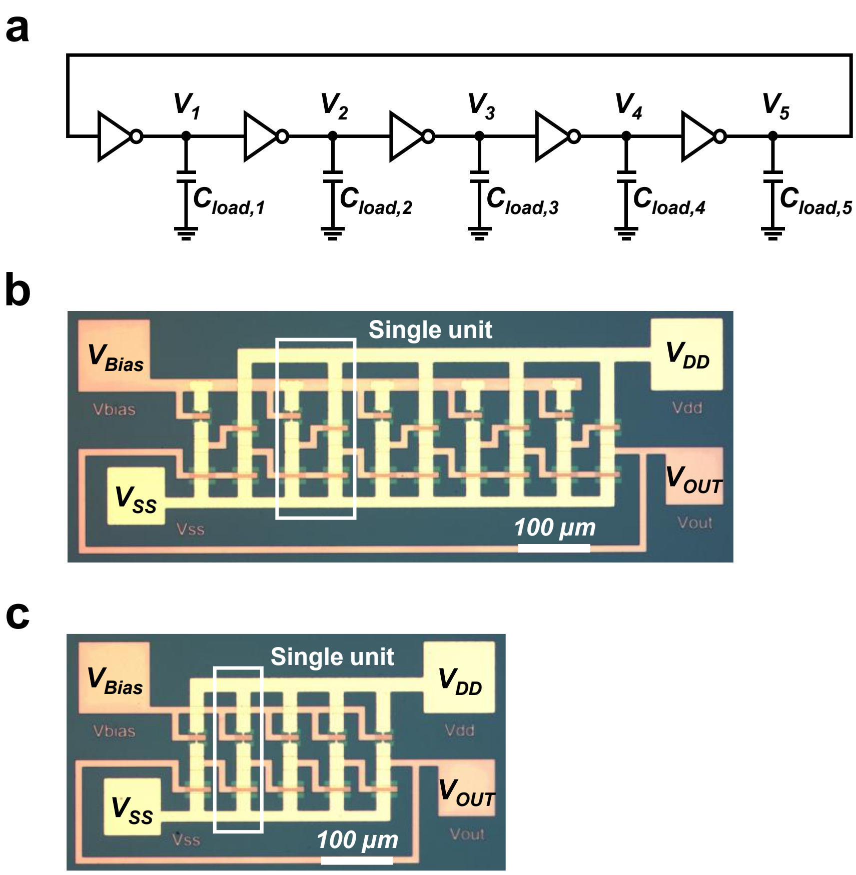

Figure S12. The schematic and optical micrographs of ROs. **a** The circuit diagram of 5-stage ring oscillator. **b** The optical micrograph of PEL inverter-based ring oscillator. **c** The optical micrograph of EL inverter-based ring oscillator.

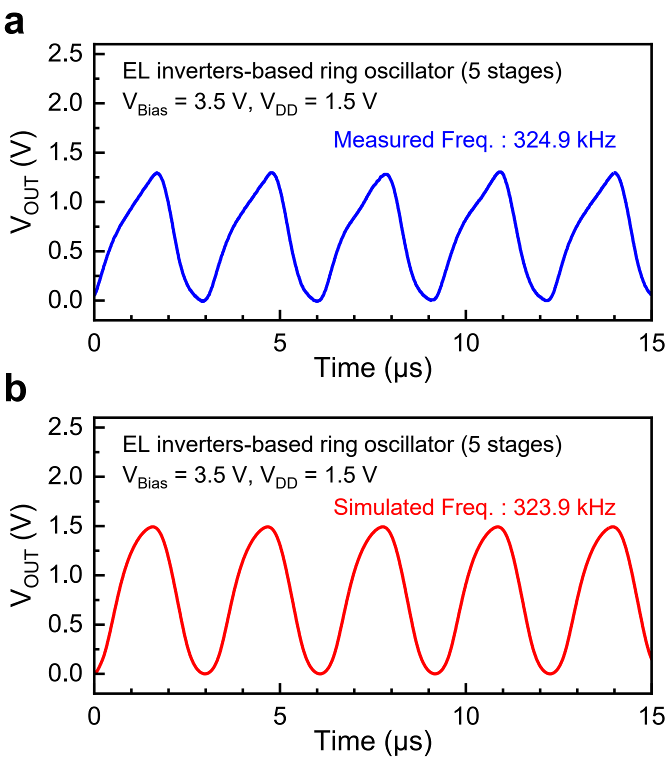

Figure S13. **a** The experimentally measured and **b** the simulated output waveforms of the EL inverter-based 5-stage ROs, with working frequency of 324.9 kHz and 323.9 kHz, respectively.

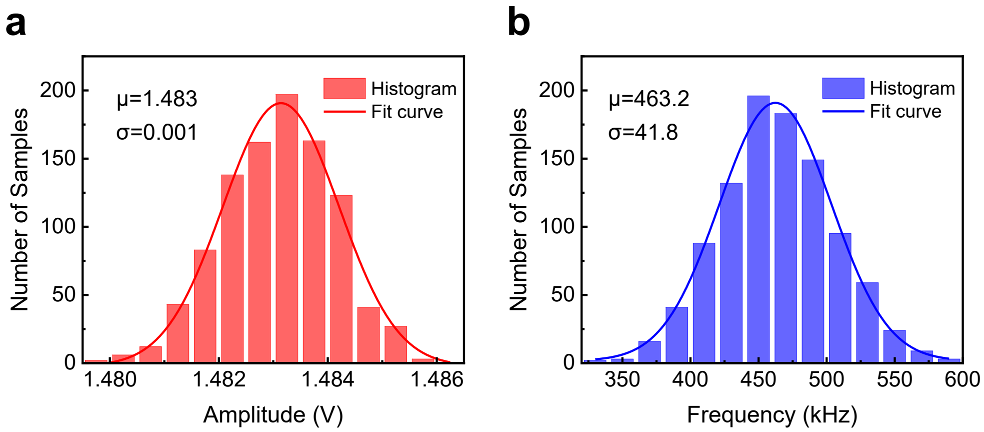

Figure S14. Monte Carlo simulations. The distribution histograms of **a** amplitude and **b** frequency of the PEL inverter-based RO obtained by performing 1000 Monte Carlo simulations.

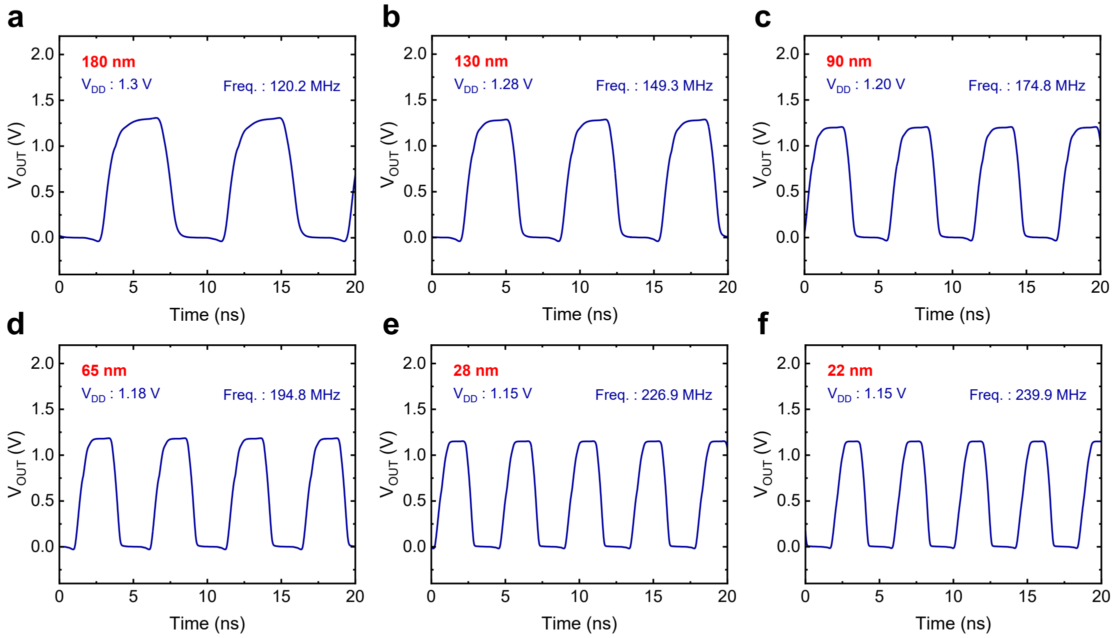

Figure S15. Projected output waveforms of ROs under various technology nodes. **a** to **f** 180 nm down to 22 nm.

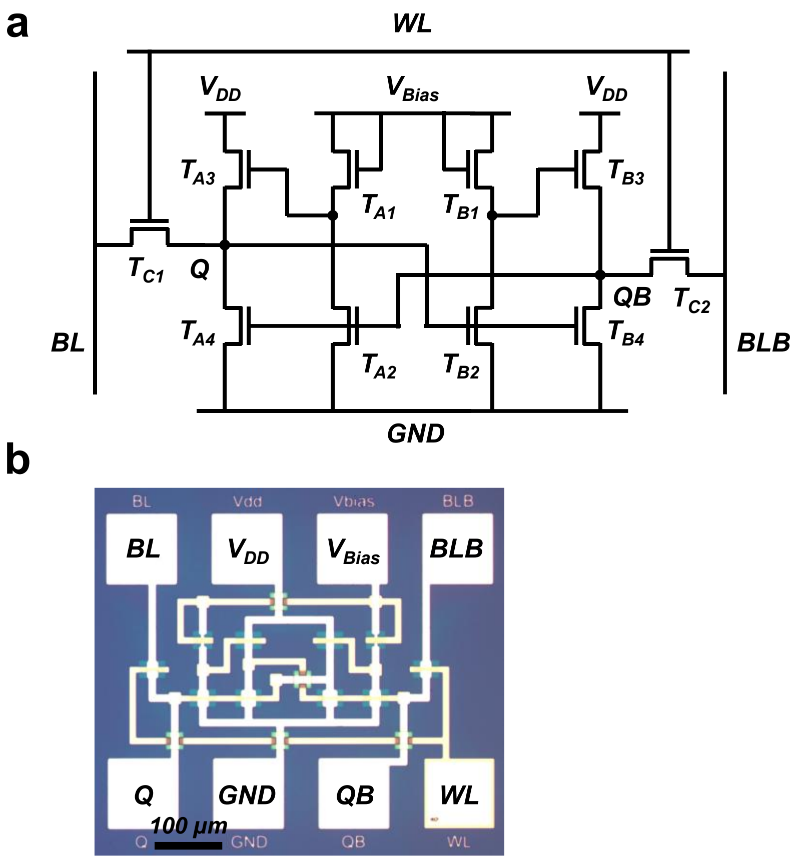

Figure S16. The schematic and optical micrograph of SRAM cell. **a** The circuit diagram of SRAM cell. **b** The optical micrograph of fabricated SRAM cell.

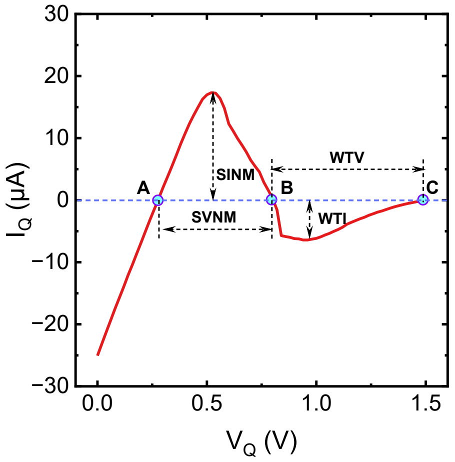

Figure S17. The N-curve of SRAM cell.

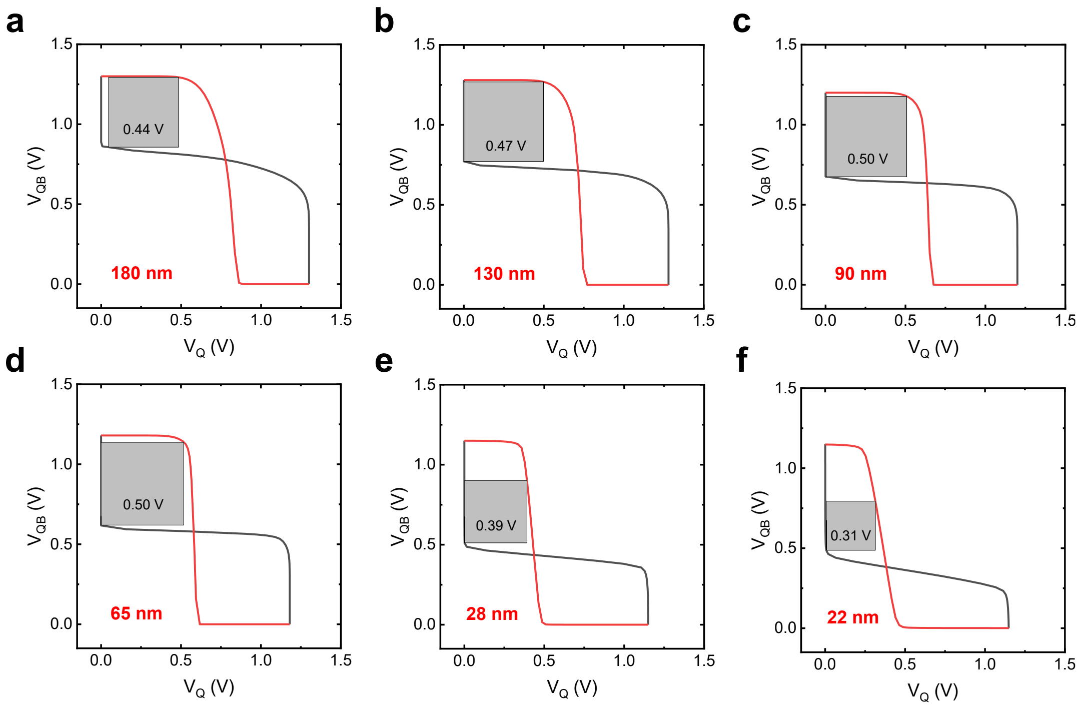

Figure S18. Simulated hold static noise margin (HSNM) at various technology nodes. **a** to **f** 180 nm down to 22 nm.

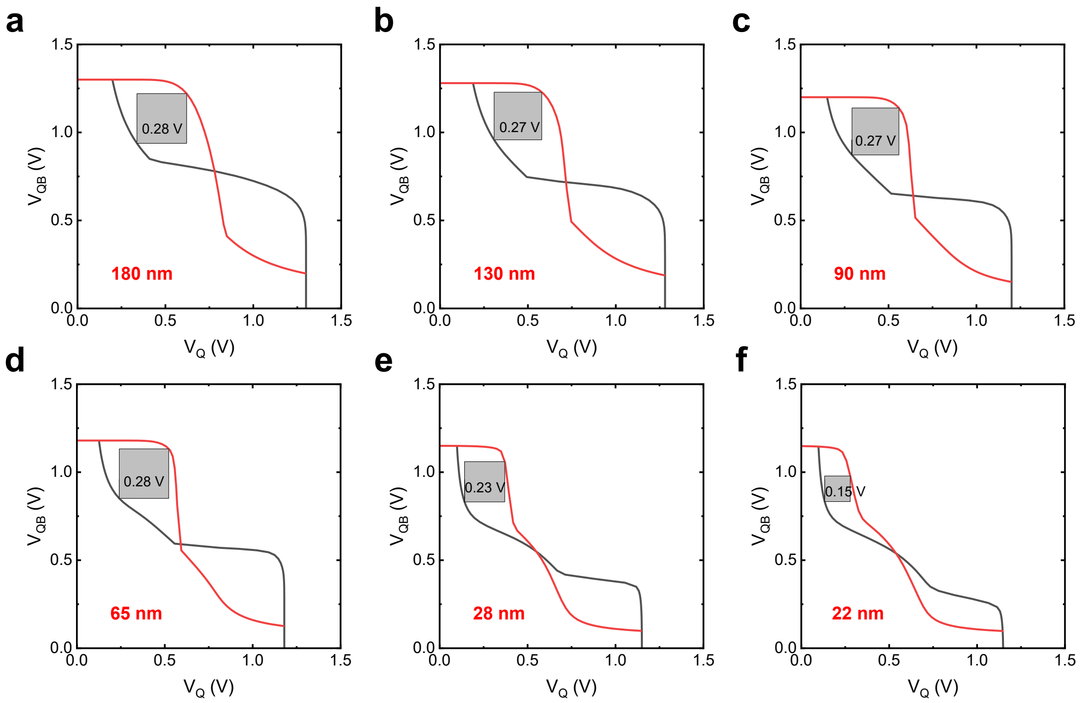

Figure S19. Simulated read static noise margin (RSNM) at various technology nodes. **a** to **f** 180 nm down to 22 nm.

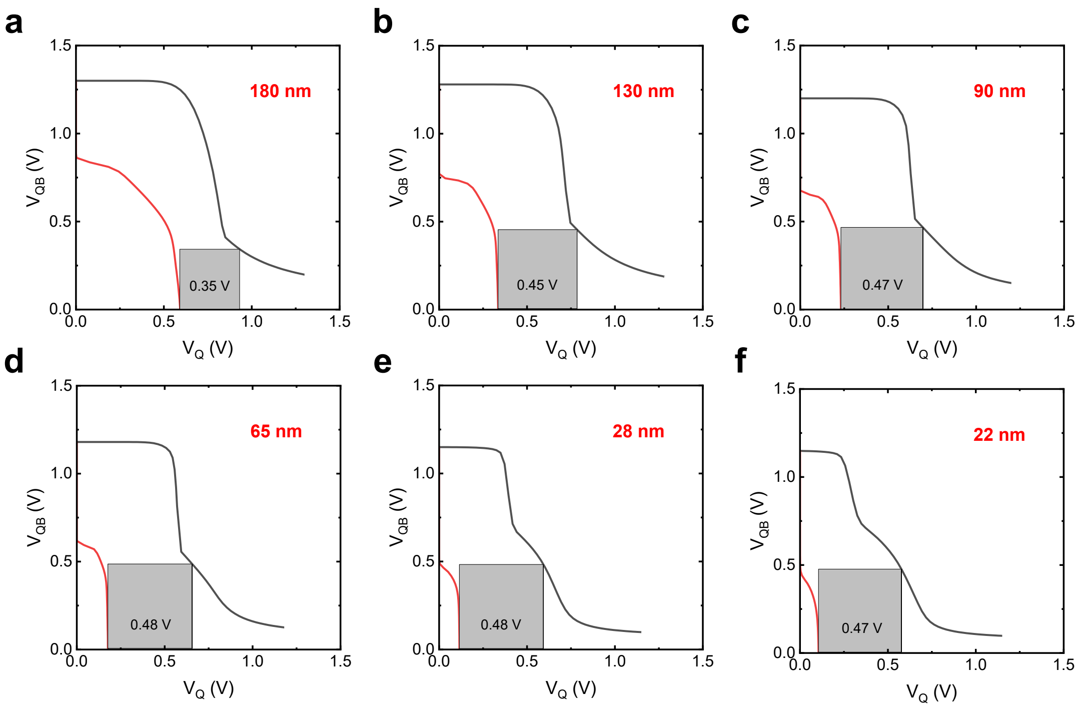

Figure S20. Simulated write static noise margin (WSNM) at various technology nodes. **a** to **f** 180 nm down to 22 nm.

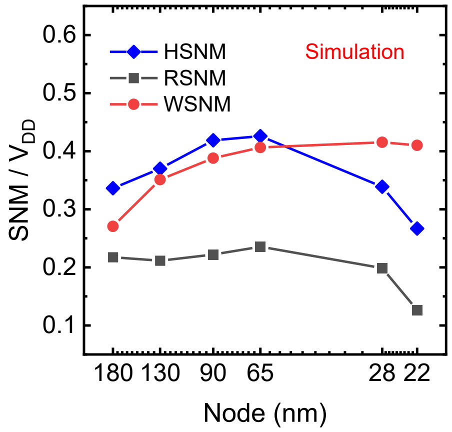

Figure S21. The summarized HSNM, RSNM, and WSNM at various technology nodes.

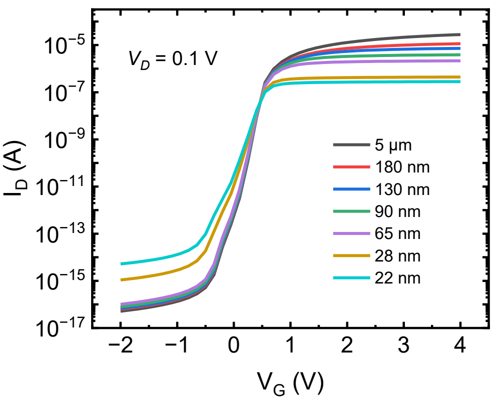

Figure S22. The simulated transfer curves of IGZO TFTs at various technology nodes. For TFT channel lengths above 65 nm, performance improvements closely follow ideal scaling; however, devices scaled below ~28 nm suffer pronounced short-channel effects that degrade subthreshold swing and induce threshold voltage drift.

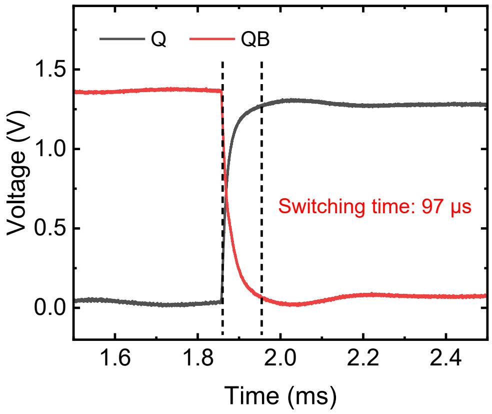

Figure S23. The measured switching time during write operation of SRAM.

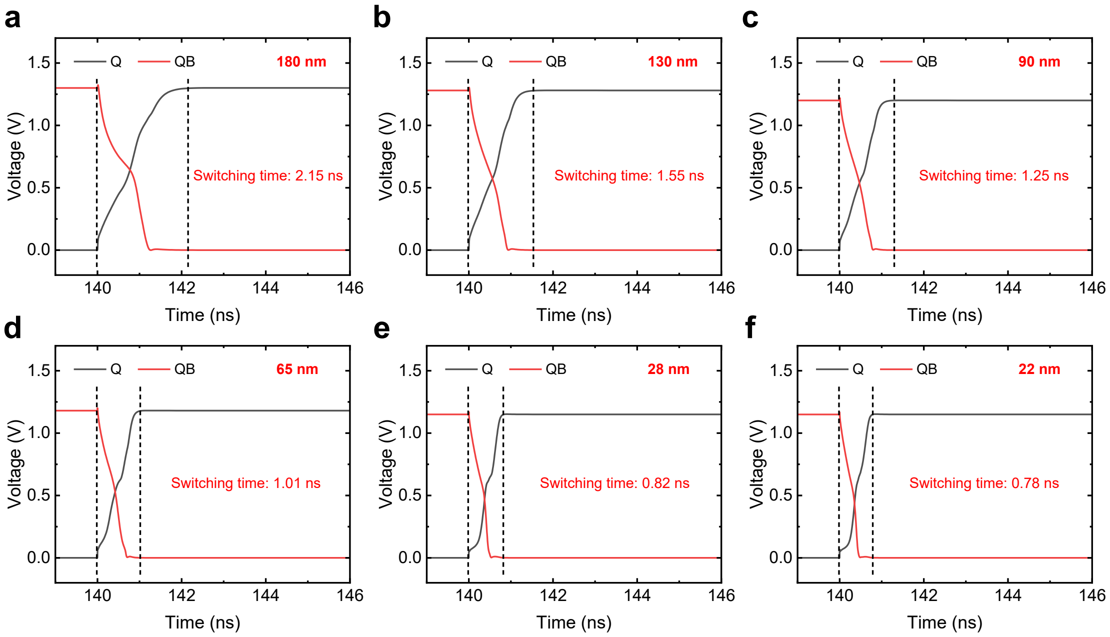

Figure S24. The switching time during write operation of SRAM at various technology nodes in simulation. **a** to **f** 180 nm down to 22 nm.

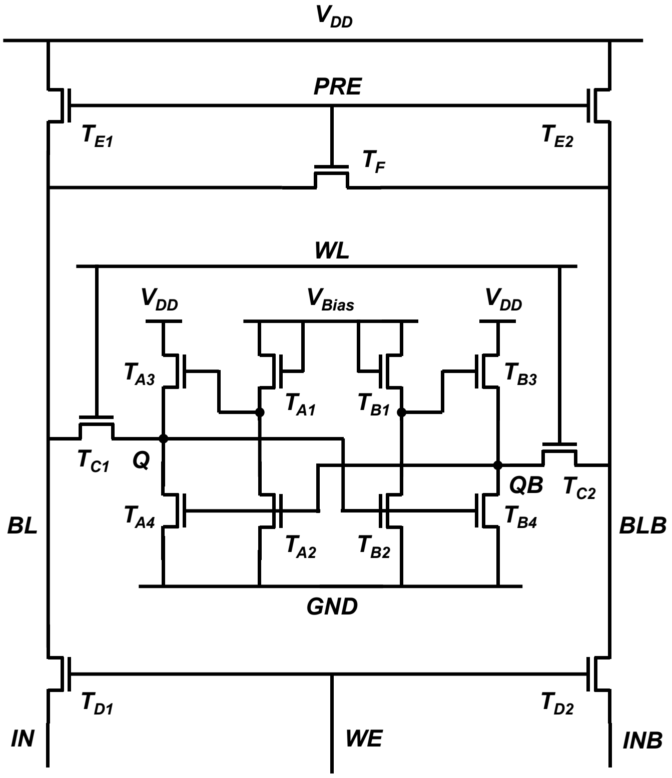

Figure S25. The circuit design for read operation of SRAM in Sentaurus.

Supporting Tables

Table S1. The geometric dimensions of the two fabricated inverters.

**Inverter type**	***T_1*** ***W_{CH} / L_{CH}*** **(μm)**	***T_2*** ***W_{CH} / L_{CH}*** **(μm)**	***T_3*** ***W_{CH} / L_{CH}*** **(μm)**	***T_4*** ***W_{CH} / L_{CH}*** **(μm)**
PEL	3 / 5	40 / 5	20 / 5	40 / 5
EL	3 / 5	40 / 5	-	-

Table S2. Performance benchmark of the two fabricated inverters.

**Inverter type**	V_{DD} **(V)**	**Gain**	**Noise margin** NM_H / NM_L **(V)**	**Rail to rail**
PEL	1.5	5.42	0.47 / 0.46	Yes
PEL	2.0	4.30	0.79 / 0.45	Yes
PEL	2.5	4.29	1.33 / 0.30	Yes
EL	1.5	2.39	0.43 / 0.10	No
EL	2.0	2.43	0.81 / -0.11	No
EL	2.5	2.48	1.14 / -0.18	No

Table S3. RO performance benchmark among this work and recently reported works.

**RO**	[1]	[2]	[3]	[4]	This work
**Materials**	IGZO	IGZO	IGZO	ITZO	IGZO
**L_{CH} (µm)**	6	10	10	10	5
**Topology**	PEL	Pseudo-C	Depletion load	Zero-V_{GS} load	PEL
**No. of stages**	13	9	3	9	5
**V_{DD} (V)**	20	6	2	10	1.5
**Voltage swing (% of V_{DD})**	-	73	-	76	88
**f_{OSC} (kHz)**	360	173.2	6.7	106	443.5
**τ_p (µs/stage)**	0.11	0.32	24.88	0.52	0.23

Table S4. The channel sizes of TFTs in SRAM cell and read simulation.

**TFTs**	W_{CH} **(μm)**	L_{CH} **(μm)**
T_{A1}, T_{B1}	3	6
T_{A2}, T_{B2}	15	5
T_{A3}, T_{B3}	10	5
T_{A4}, T_{B4}	20	5
T_{C1}, T_{C2}	15	5
T_{D1}, T_{D2}	25	5
T_{E1}, T_{E2}	20	5
T_F	40	5

Table S5. Performance benchmark of the state-of-the-art SRAM.

Ref	Technology	Area (mm^2)	V_{DD} (V)	HSNM (V)	Normalized HSNM	RSNM (V)	WSNM (V)
[5]	CNT	0.5	5	0.9	36.0%	-	-
[6]	CNT	0.6	1.75	-	-	0.32	0.37
[7]	Organic	> 1	40	-	-	-	-
[8]	IGZO	0.03	15	2.57	34.3%	2.37	-
[9]	IGZO-SnO	0.02	8	2.30	57.5%	1.43	1.67
[10]	Organic	2.1	3	0.76	50.7%	0.64	0.70
[11]	IGZO-CNT	0.64	5	1.84	73.6%	1.07	0.92
This work	IGZO	0.08	1.5	0.45	60.0%	0.26	0.25

Table S6. The relevant material parameters used in TCAD modelling.

Parameter	IGZO	HfO_2
Effective density of states of the conduction band (N_C)	5×10^{18} $cm^{-3} \cdot eV^{-1}$	-
Effective density of states of the valence band (N_V)	5×10^{18} $cm^{-3} \cdot eV^{-1}$	-
Bandgap	3.53 eV	-
Electron affinity	4.20 eV	-
Reference mobility used in doping-dependent mobility model	45.94 $cm^2/V \cdot s$	-
Dielectric constant	-	15

Table S7. Supply voltages and load capacitor values used for device performance projection.

Node (nm)	1500	180	130	90	65	28	22
V_{DD} (V)	1.50	1.30	1.28	1.20	1.18	1.15	1.15
C_{load} (fF)	15400	19.96	10.41	4.99	2.60	0.48	0.30

Supporting Notes

Note S1. The extraction methods for V_{TH}, μ_{FE}, and SS

<u>The extraction method for V_{TH}</u>

We utilized linear extrapolation method to extract the threshold voltage (V_{TH}) in this work.[12] The V_{TH} was extracted from transfer curves (I_D-V_G) of TFTs at low drain voltage (V_D = 0.1 V) to ensure TFT's operation in the linear region. The value of V_{TH} is determined from V_G axis intercept (I_D = 0 A) of the linear extrapolation of I_D-V_G curve at the maximum slope point, as illustrated in **Figure S26**.

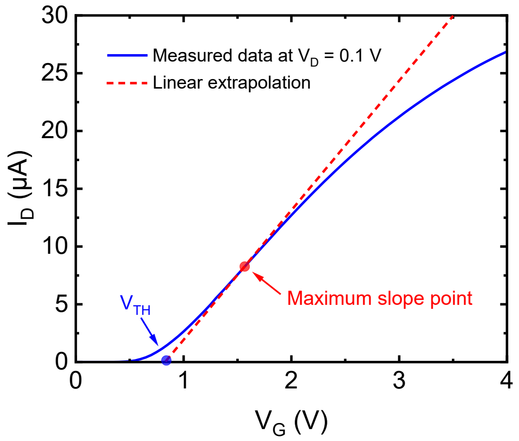

Figure S26. The linear extrapolation method implemented on transfer curve (I_D-V_G) at low drain voltage (V_D = 0.1 V).

<u>The extraction method for μ_{FE}</u>

The field-effect mobility (μ_{FE}) was obtained using the maximum transconductance (g_m) from the transfer curves (I_D-V_G).[13] The calculation equations for g_m and μ_{FE} are

$$g_m = \frac{\partial I_D}{\partial V_G} \tag{1}$$

$$\mu_{FE} = \frac{g_m}{C_i \frac{W_{CH}}{L_{CH}} V_D} \tag{2}$$

, where C_i is the unit-area capacitance of gate dielectric ($C_i = \varepsilon \cdot \varepsilon_0 / T_{ox}$, where ε is relative permittivity of HfO_2 dielectric, ε_0 is vacuum permittivity, and T_{ox} is the thickness of HfO_2), W_{CH} and L_{CH} are the channel width (10 μm) and length (5 μm) of TFT respectively, and V_D is the drain voltage (V_D = 0.1 V).

<u>The extraction method for SS</u>

The subthreshold swing (SS) was also extracted from transfer curves (I_D-V_G) of TFTs at low drain voltage (V_D = 0.1 V) and calculated using following equation [14-15]:

$$SS = \frac{\partial V_G}{\partial \log (I_D)} \tag{3}$$

, where V_G is the gate voltage, and I_D is the drain current.

Note S2. The calculation method for bandgap

We utilized Tauc plot method,[16] to calculate the bandgaps of IGZO films with different cation ratios in this work. This method is mainly based on the following equation:

$$(\alpha h v)^{1/n} = B(hv - E_g) \quad (4)$$

, where α is absorption coefficient, h is Planck constant, v is incident photon frequency, B is proportional constant, and E_g is bandgap. The index n is related to the type of semiconductor material, with $n = 1/2$ for a direct bandgap and $n = 2$ for an indirect bandgap.

Here, we replaced the α with the absorbance (Abs) in calculation, because Abs is directly proportional to α.

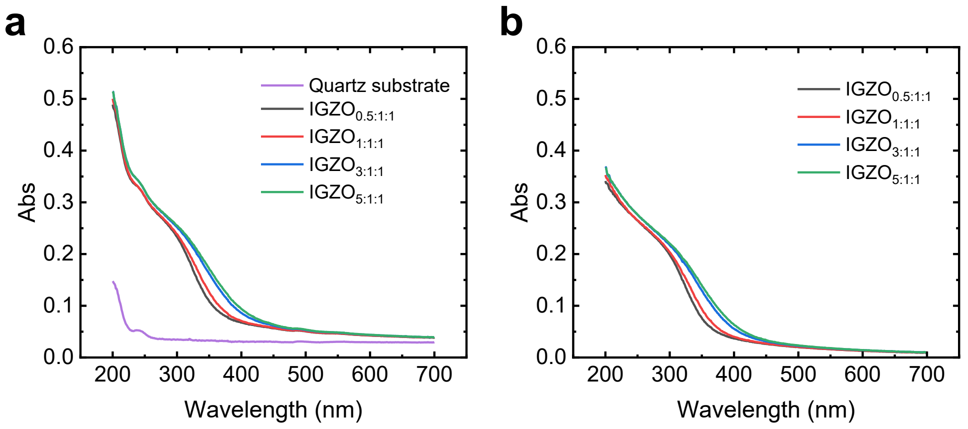

Figure S27. a The measured absorbance as a function of wavelength of IGZO films with different cation ratios on quartz substrate and of quartz substrate alone. **b** The absorbance of IGZO films subtracting that of the quartz substrate.

The IGZO films with different cation ratios were deposited on the quart substrate for bandgap measurement. For precise analysis, we measured the absorbance of both the IGZO films on the quartz substrate and the quart substrate alone using an UV-Vis-NIR Spectrometer under the same conditions (**Figure S27a**). Then, we subtracted the absorbance of the quartz substrate from the absorbance of the IGZO films plus substrate, to get the absorbance solely due to the IGZO films (**Figure S27b**).

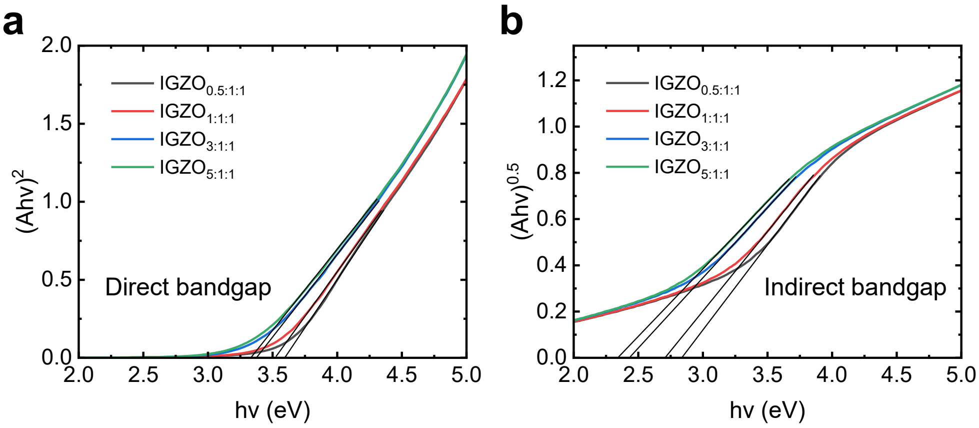

Figure S28. a, **b** The illustration of direct bandgap and indirect bandgap calculation method.

For direct bandgap,

$$(Ahv)^2 = C(hv - E_g) \tag{5}$$

, where A is the absorbance and C is a constant. The hv could be determined by the equation of $hv = 1240/\lambda$, where λ is the incident photon wavelength. Plot the $(Ahv)^2$ as the function of hv, and the direct bandgap value is determined from hv axis intercept ($(Ahv)^2$ = 0) of the linear extrapolation of $(Ahv)^2 - hv$ curve during its linear region, as illustrated in **Figure S28a**.

For indirect bandgap,

$$(Ahv)^{0.5} = C(hv - E_g) \tag{6}$$

Plot the $(Ahv)^{0.5}$ as the function of hv, and the indirect bandgap value is determined from hv axis intercept ($(Ahv)^{0.5}$ = 0) of the linear extrapolation of $(Ahv)^{0.5} - hv$ curve during its linear region, as illustrated in **Figure S28b**.

Note S3. The evaluation method for D_{it}

We utilized the high- and low-frequency capacitance measurements (Castagné-Vapaille method),[17] to evaluate the interface state density (D_{it}) in this work.

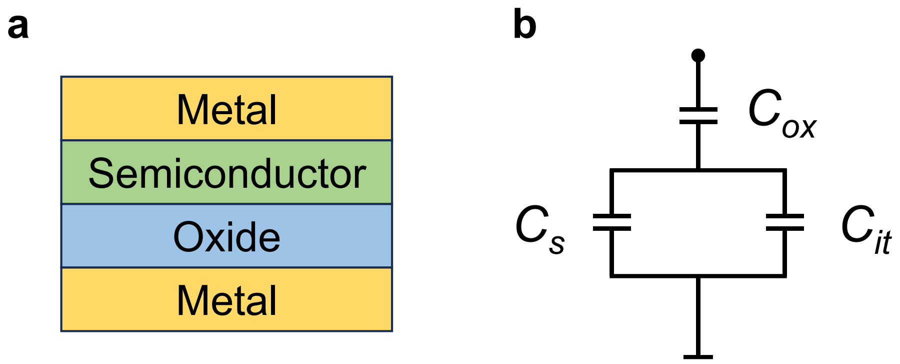

Figure S29. a The MOSCAP structure and **b** the equivalent circuit of the MOSCAP in depletion region.

We fabricated a MOSCAP structure to measure the capacitance-voltage (C-V) curves (**Figure S29a**), and the fabrication process of MOSCAPs was same as that of TFT. The equivalent circuit of MOSCAPs consists of the oxide capacitance (C_{ox}), the semiconductor capacitance (C_s), and the equivalent parallel interface trap capacitance (C_{it}), as illustrated in **Figure S29b**. The total capacitance (C_{tot}) of the MOSCAP is given by:

$$\frac{1}{C_{tot}} = \frac{1}{C_{ox}} + \frac{1}{C_s + C_{it}} \tag{7}$$

The C_{it} can be determined by measuring the total capacitance at a sufficiently low frequency (C_{lf}) so that the interface traps can follow the AC signal. The D_{it} can be determined by C_{it}, and is given by:

$$D_{it} = \frac{C_{it}}{qA} \tag{8}$$

, where A is the area of MOSCAP, and so, the D_{it} can be expressed by:

$$D_{it} = \frac{1}{qA}\left(\frac{C_{ox}C_{lf}}{C_{ox} - C_{lf}} - C_s\right) \tag{9}$$

The C_s can be deduced from the total capacitance measured at a sufficiently high frequency (C_{hf}), so that the interface traps cannot respond to the AC signal, namely the $C_{it} \approx 0$. Under such high-frequency measurement conditions, the total capacitance equation (**Equation (7)**) can be expressed as:

$$\frac{1}{C_{hf}} = \frac{1}{C_{ox}} + \frac{1}{C_s} \tag{10}$$

Combining **Equation (9)** and **Equation (10)**, the D_{it} is given by:

$$D_{it} = \frac{1}{qA}\left(\frac{C_{ox}C_{lf}}{C_{ox} - C_{lf}} - \frac{C_{ox}C_{hf}}{C_{ox} - C_{hf}}\right) \tag{11}$$

To map the D_{it} values from the gate voltage (V_G) domain to the energy domain, the relationship between surface potential ψs and V_G was established by applying the Berglund integral to the C_{lf} curve. For this analysis of our IGZO n-type device, the reference voltage (V_{ref}) for the integral was selected as the maximum applied voltage, corresponding to the strong accumulation regime. This reference point E_{ref} is physically

defined as the energy level where the Fermi level (E_f) is pinned near the conduction band edge (E_c). The integral is thus:

$$\psi_s(V_G) = \int_{Vref}^{V_G}(1 - \frac{C_{lf}(V')}{C_{ox}})dV' + \psi_{s,ref} \quad (12)$$

Then, the V_G-dependent energy can be obtained by $E(V_G) = q\psi_s(V_G)$.

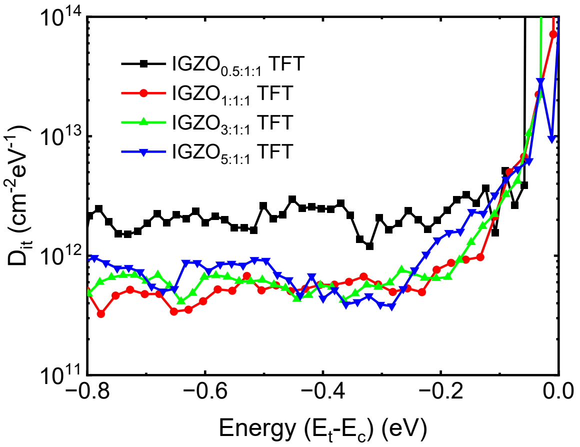

Figure S30. The energy dependent D_{it} calculated from C-V data for IGZO TFTs with various In content.

Figure S30 shows the energy-dependent D_{it} calculated from the C-V characteristics of IGZO devices with different In contents. The $IGZO_{0.5:1:1}$ device exhibits the highest D_{it} over most of the energy range, whereas the $IGZO_{1:1:1}$ device presents the lowest and most uniform D_{it}, which is consistent with the *SS* results ($IGZO_{0.5:1:1}$ TFT has the largest *SS*, while $IGZO_{1:1:1}$ TFT has the smallest *SS*). Moreover, the D_{it} levels of $IGZO_{1:1:1}$, $IGZO_{3:1:1}$ and $IGZO_{5:1:1}$ device are comparable, suggesting that interface/scattering traps are not the primary factor limiting mobility. This further indicates that the In content predominately governs the carrier transport, enabling the highest mobility to be obtained in the high-In device ($IGZO_{5:1:1}$).

Note S4. The performance comparisons between the PEALD and sputtered IGZO TFTs

We conducted additional experiments on sputtered IGZO TFTs, which are identical in device architecture, gate dielectric, contact metallization, and thermal treatments to the PEALD devices. The sputtering conditions for the IGZO channel layer were: 4 mTorr chamber pressure, 80 W RF power, 300 s deposition time, using a 1:1:1 In: Ga: Zn oxide target.

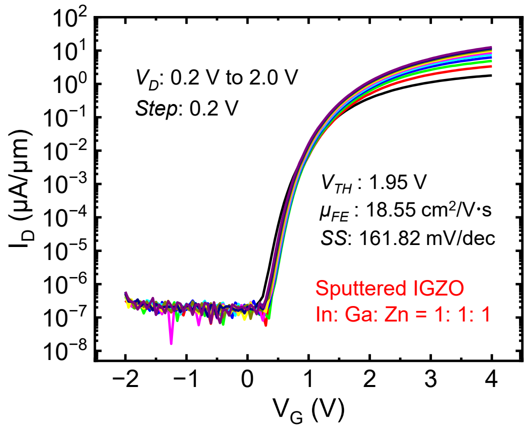

Figure S31. The transfer characteristics of the sputtered IGZO TFTs, labelled with key electrical performance metrics.

Table S8. The key electrical parameters comparison between PEALD IGZO TFTs and sputtered IGZO TFTs.

Parameter	Sputtered IGZO TFTs	PEALD IGZO TFTs
V_{TH} (V)	1.95 ± 0.26	0.84 ± 0.09
μ_{FE} (cm^2/V·s)	18.55 ± 2.01	39.17 ± 2.21
SS (mV/dec)	161.82 ± 32.56	74.60 ± 4.27

Figure S31 shows the transfer characteristics of the sputtered IGZO TFTs, with key electrical performance metrics compared with PEALD IGZO TFTs in **Table S8**. In comparison, the sputtered IGZO TFTs exhibit a lower field-effect mobility (μ_{FE}) of 18.55 cm^2/V·s versus 39.17 cm^2/V·s for PEALD devices, and a broader subthreshold swing (*SS*) of 161.82 mV/dec versus 74.60 mV/dec. These results confirm that, under the same process flow and device geometry, PEALD process yields a denser, lower-defect channel, which translates into higher mobility and faster switching, thereby demonstrating the clear performance advantages of the PEALD IGZO deposition process.

Note S5. The effect of PEALD process parameters on IGZO TFT performance

We systematically investigated the influence of PEALD process parameters (deposition temperature, plasma power, and precursor pulse/purge time) on the electrical properties of IGZO TFTs.

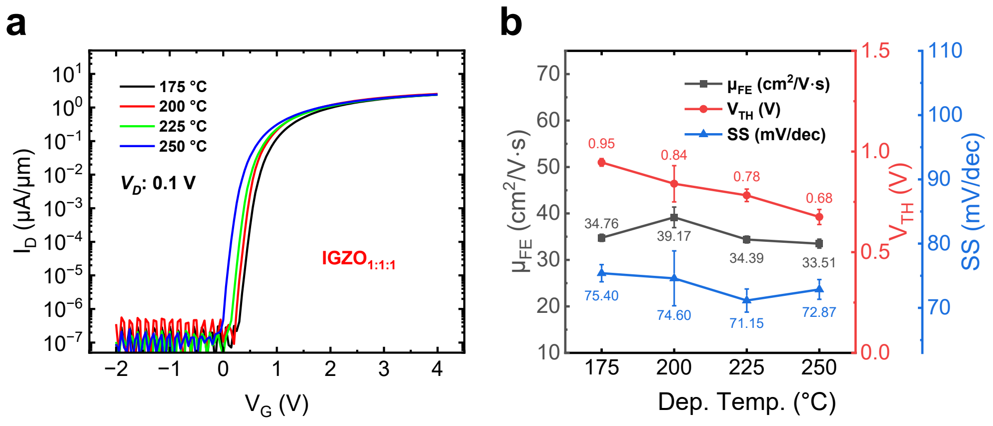

Figure S32. **a** The transfer characteristics of fabricated $IGZO_{1:1:1}$ TFTs under different deposition temperatures. **b** The corresponding extracted V_{TH}, μ_{FE}, and SS with error bar.

We investigated the influence of IGZO deposition temperature on TFT performance. **Figure S32a** presents the transfer characteristics of $IGZO_{1:1:1}$ TFTs deposited at temperatures ranging from 175 to 250 °C. The corresponding extracted V_{TH}, μ_{FE}, and SS together with error bar are plotted in **Figure S32b**. It is observed that the increase in the deposition temperature results in a decrease in the V_{TH}. This is likely attributed to a reduction in defects concentration in the IGZO film due to the higher temperature, resulting in the reduction of the bandgap.[18]

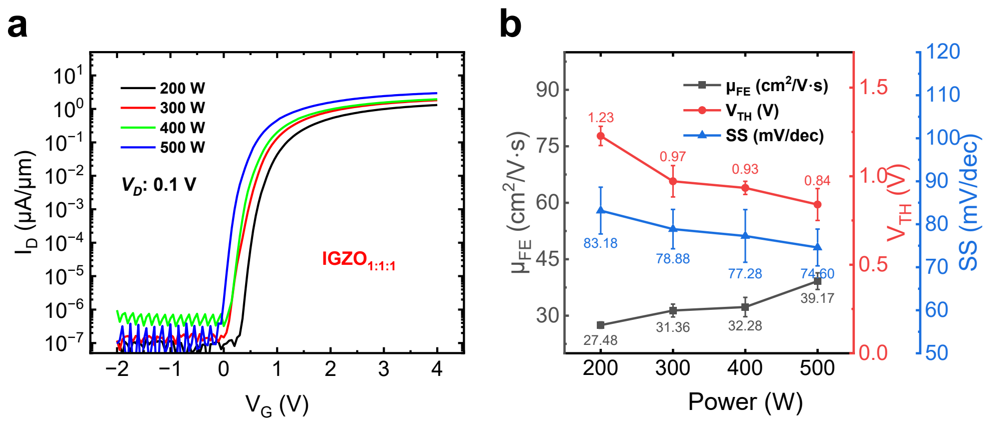

Figure S33. **a** The transfer characteristics of fabricated $IGZO_{1:1:1}$ TFTs under different PEALD power. **b** The corresponding extracted V_{TH}, μ_{FE}, and SS with error bar.

We further evaluated the influence of plasma power during PEALD on TFT performance. **Figure S33a** shows the transfer characteristics for devices deposited at powers from 200 to 500 W, and **Figure S33b** summarizes the extracted V_{TH}, μ_{FE}, and SS with error bars. Increasing the PEALD power yields higher μ_{FE} and lower SS, accompanied by a negative shift in V_{TH}. These trends are consistent with enhanced plasma reactivity at elevated power, which promotes more complete surface reactions and film densification, thereby

reducing trap density and carrier scattering; a modest increase in donor-like defects (e.g., oxygen vacancies) may further contribute to the observed V_{TH} shift.

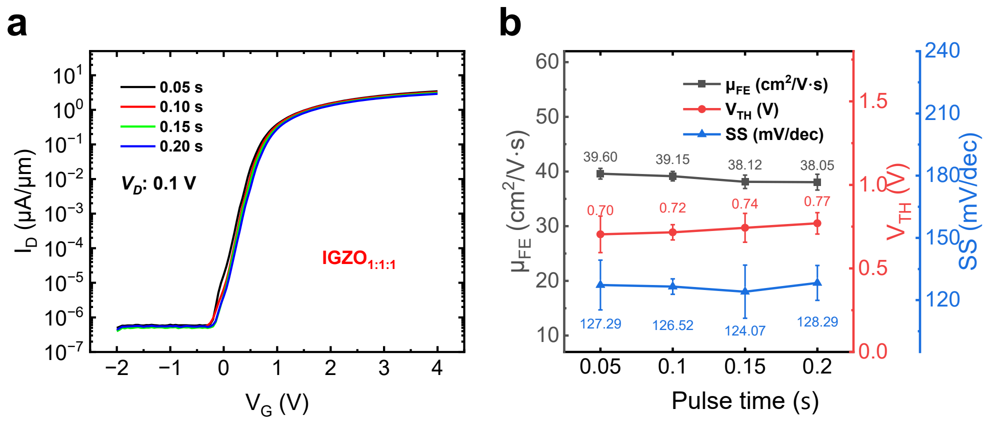

Figure S34. **a** The transfer characteristics of fabricated $IGZO_{1:1:1}$ TFTs under different pulse time during PEALD process. **b** The corresponding extracted V_{TH}, μ_{FE}, and SS with error bar.

To assess the impact of pulse time during PEALD process on device behavior, we examined IGZO films deposited with pulse times spanning from 0.05 to 0.20 s. The transfer characteristics are shown in **Figure S34a**, and the extracted V_{TH}, μ_{FE}, and SS, summarized with error bars in **Figure S34b**, exhibit negligible dependence on pulse time within this range. This invariance is consistent with the self-limiting, layer-by-layer growth inherent to ALD. Likewise, variations in purge time during PEALD also predictably produced no measurable changes in the TFT electrical characteristics.

Note S6. The evaluation of contact resistance

To evaluate the contact resistance (R_C) and investigate its effect on mobility of the fabricated devices, we characterized TFTs with different channel length (L_{CH}), namely employing transfer length method (TLM).

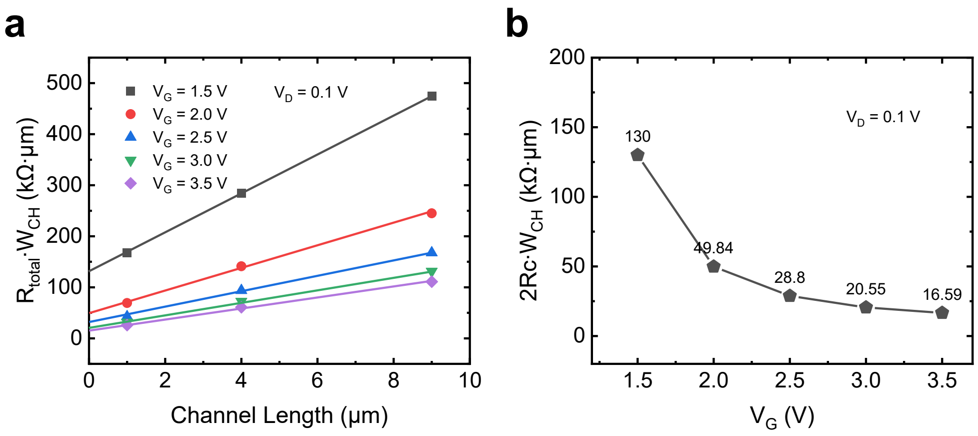

Figure S35. **a** The channel width (W_{CH}) normalized total resistance (R_{total}) of IGZO TFTs with varying channel length (L_{CH}). **b** The W_{CH}-normalized contact resistance (R_C) versus gate voltage (V_G) through transfer length method (TLM).

Figure S35a plots the total resistance (R_{total}) normalized by channel width (W_{CH}), i.e., $R_{total}\cdot W_{CH}$, as a function of L_{CH} for the IGZO TFTs. As the gate voltage (V_G) increases, the channel enters stronger conduction, resulting in reduced slope and intercept of the $R_{total}\cdot W_{CH}$ versus L_{CH} curves. The y-axis intercept of $R_{total}\cdot W_{CH}$ corresponds to the W_{CH}-normalized R_C (i.e., $2R_C\cdot W_{CH}$), as shown in **Figure S35b**.

Furthermore, we calculated the intrinsic channel mobility μ_o (excluding the influence of R_C) using following equation:

$$\mu_o = \frac{\mu_{FE}}{1-\frac{2R_C}{R_{total}}} \tag{13}$$

μ_o is defined as the mobility of the IGZO channel in the absence of R_C, while μ_{FE} represents the effective mobility calculated in the presence of R_C. We obtained μ_o = 72.04 cm^2/V·s, which is substantially higher than the μ_{FE} of 39.17 cm^2/V·s, confirming that R_C markedly limits device mobility. Accordingly, future efforts should focus on contact engineering—such as surface plasma treatments and doped contact regions—to minimize R_C and further enhance mobility.

Note S7. Strategies to mitigate the V_{TH} shift in high-mobility compositions

The concentration of indium (In) in the IGZO films strongly influences the density of oxygen vacancies (V_O) and free carriers. In's large 5s orbitals form the conduction band minimum, enabling higher electron mobility and percolative transport even in disordered amorphous structure. Thus, In-rich IGZO films typically show enhanced mobility and carrier density. However, In-rich compositions are prone to more oxygen vacancies, which act as shallow donor defects. These excess donors push the Fermi level closer to the conduction band, resulting in a negatively shifted V_{TH} or even a normally-on transistor in the as-fabricated state. Thus, it is difficult to achieve high carrier mobility without inducing a negative V_{TH} shift merely through tuning the cation ratios in IGZO. However, some stack and device structure strategies may mitigate the V_{TH} shift in high-mobility IGZO TFTs.

Dual-active-layer channels: One proven strategy is using a bilayer channel, typically combining an In-rich layer (for high mobility) with a Ga-rich or dopant-rich layer (for stability and low off-current).

Channel layer arrangement and thickness: Even within a mono IGZO layer, researchers have found that controlling the vertical atomic arrangement can help. For instance, Choi et al. (2025) report that increasing the thickness of the In sublayer within a fixed cycle not only boosts mobility but also counters the negative shift in V_{TH}, demonstrating the precise atomic-scale arrangement within the IGZO films influences both device performance and stability.[19]

Gate stack and dielectric engineering: The gate insulator and its interface with IGZO also play a role in the mobility-stability balance. High-k dielectrics (like HfO_2) are often used to enhance capacitance and reduce operating voltage, but they can induce additional charge trapping. One strategy has been inserting an ultra-thin interfacial layer between IGZO and a high-k gate dielectric (for example, a few nanometers of SiO_2 or an Al_2O_3 "seed" layer) to improve interface quality. This can lead to a more positive and stable V_{TH} without severely diminishing mobility.

Dual-gate structure: Dual-gate control is another lever, namely top/bottom-gate biasing screens the back channel and reduces V_{TH} drift without compromising mobility.

Note S8. The evaluation methods and analyses for HSNM, RSNM, WSNM and N-curve

To assess the HSNM, the *WL* was set to 0 V to deactivate the access transistors, while both *BL* and *BLB* were held at V_{DD}. A voltage sweep was applied from 0 V to V_{DD} at the *Q* node, and the voltage at the *QR* node was recorded.[9] With V_{DD} set at 1.5 V, the measured voltage transfer characteristic (VTC) curve and its mirror curve (reflected about the 45° line) are presented in **Figure 6a** (in main text). The analysis reveals a high HSNM value of 0.45 V, which corresponds to 60% when normalized by $V_{DD}/2$, equating to the side length of the inserted square. This high noise margin indicates excellent stability of the SRAM cell in its data hold state.

For evaluating the read operation, the RSNM was characterized by connecting *WL*, *BL*, and *BLB* to V_{DD} (1.5 V) and sweeping the voltage at the *Q* node while monitoring the *QB* node. As shown in **Figure 6b** (in main text), the resulting VTC curve and its mirrored counterpart yield a RSNM value of 0.26 V (34.67%, normalized by $V_{DD}/2$). The intersection points, labeled as A, B, and C, denote the three operational states during a read cycle: points A and C represent the two stable states (corresponding to logic “0” with $V_Q = 0.17$ V and logic “1” with $V_Q = 1.5$ V, respectively), while point B corresponds to a metastable state (at $V_Q = 0.75$ V). This behavior confirms that the cell can reliably output one-bit binary data during read operations.

The write capability was similarly analyzed through the WSNM measurement. During a write operation, *WL* and *BL* were connected to V_{DD}, while the *BLB* was set to V_{DD} for writing a “1” and to 0 V for writing a “0”. As depicted in **Figure 6c** (in main text), the SRAM cell exhibits a WSNM value of 0.25 V (33.33%, normalized by $V_{DD}/2$), demonstrating that one-bit binary data can be stably written into the cell.

Additionally, the N-curve method was also employed to further evaluate the read/write performance.[20] This method illustrates the relation between the current and voltage at the internal node *Q* when *WL*, *BL*, and *BLB* are maintained at V_{DD}, as shown in **Figure S17**. Here, three intersection points at $I_Q = 0$ A are observed: two points corresponding to stable states (A and C) and one point corresponding to a metastable state (B). From this analysis, a static voltage noise margin (SVNM) of 0.52 V and a static current noise margin (SINM) of 17.33 μA were extracted. These values represent the maximum DC noise voltage and current tolerances at node *Q*, respectively. Furthermore, the write-trip voltage (WTV) and write-trip current (WTI) were determined to be 0.70 V and -6.42 μA, respectively, providing additional insight into the write stability of the cell.

Although only n-type TFTs were used in the SRAM, all the static characteristics, including HSNM, RSNM, and WSNM, exhibited large values and good robustness, which can be attributed to the high performance of optimized IGZO TFTs and pseudo-CMOS structure of inverters. This provides insights for the unipolar circuit design based on oxide semiconductors.

Note S9. The continuous write and read timing illustration

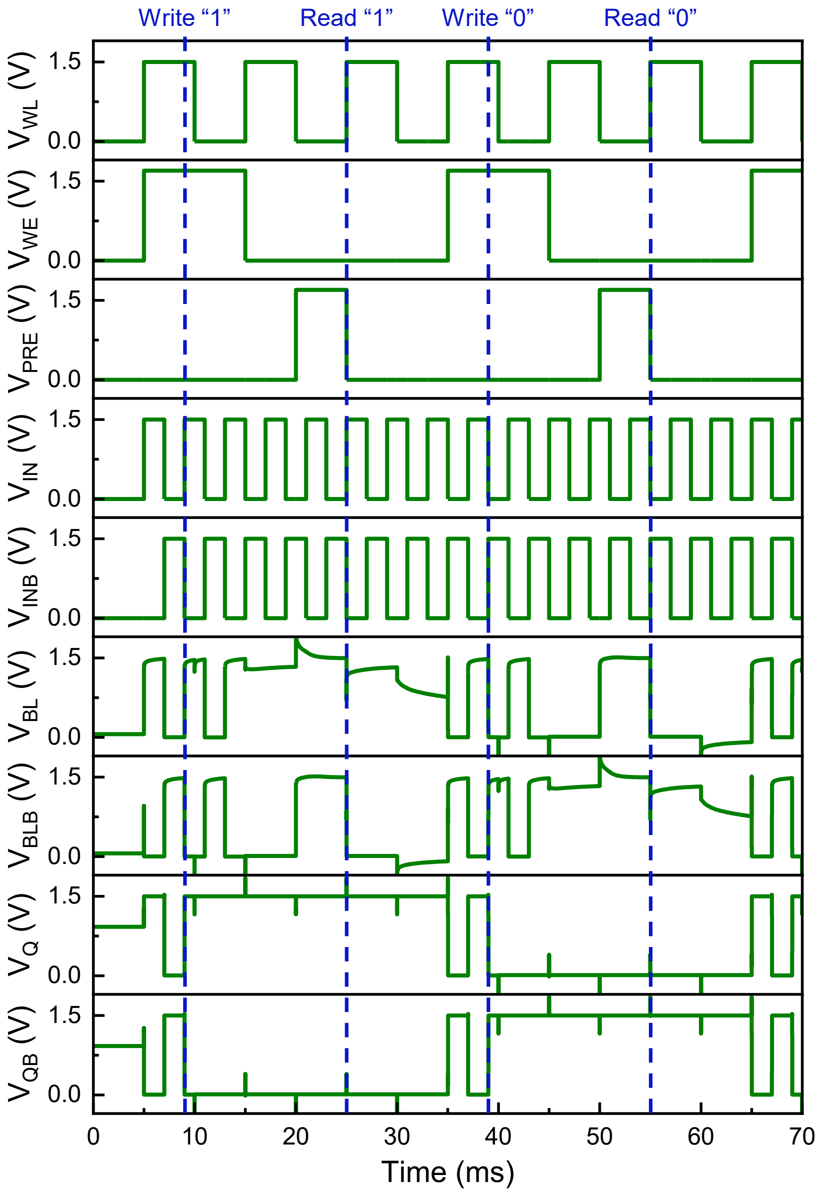

Figure S36. The simulated timing sequence in continuous write and read operation.

The simulated timing sequence in continuous write and read operation at all ports is shown in **Figure S36**, and proceeded as follows.

During the write operation, the write-enable (*WE*) signal remained high to activate the write-driver circuit, while the pre-charge (*PRE*) signal was held low to isolate the pre-charge circuit, thereby preventing interference with the write process. The complementary inputs, *IN* and *INB*, set the desired logic levels on the bit lines (*BL* and *BLB*), and their timing relationship to the word line (*WL*) mirrored that of a conventional write operation. After completing the write operation, the *WE* signal was de-asserted to disable the write driver, and the *PRE* signal was raised to pre-charge the *BL* and *BLB*. When *WL* was asserted high, the access transistors were activated, allowing *BL* and *BLB* to sense the stored values at *Q* and *QB*.

Note S10. The limitations in scaling PEALD IGZO TFTs for dense logic circuits

When scaling PEALD IGZO TFTs toward high-density logic integration, several device-level and circuit-level challenges emerge.

Parasitic capacitance becomes a major bottleneck as channel lengths shrink below 100 nm. As devices shrink, the relative impact of gate-to-source/drain overlap capacitances becomes pronounced. This fundamental issue stems from the difficulty in implementing self-aligned architectures common in silicon CMOS. This significantly increases overlap capacitances – the most critical parasitic component – which dominates switching delays as dimensions scale. Furthermore, fringe capacitance intensifies aggressively at ultra-scaled nodes (<50 nm), exacerbated by the necessary use of high-k gate dielectrics. These dielectrics enhance electrostatic control but amplify lateral electric field coupling between the gate and adjacent electrodes.

Self-heating presents a critical limitation due to IGZO's relatively poor thermal conductivity (~1 W/m·K – orders of magnitude lower than silicon). As transistors shrink and packing density increases, heat generated by Joule dissipation becomes highly localized within the channel. Dense interconnect structures in logic circuits further exacerbate heating through thermal crosstalk, creating localized hotspots. Moreover, repeated thermal cycling can accelerate defect generation in the gate dielectric and at the IGZO interface, leading to threshold voltage drift and long-term reliability concerns, especially under the tight thermal budgets of dense logic fabrics.

Contact resistance (R_C) rapidly becomes the dominant resistance component as the channel length scales below approximately 100 nm. As channel lengths decrease, the proportion of voltage drop and series resistance contributed by source/drain contact regions and metal-semiconductor junctions grows, limiting the overall on-state current and increasing propagation delays. Achieving required R_C values below 10^{-7} $\Omega\cdot cm^2$, necessary for high-performance logic at sub-100 nm nodes, remains elusive in IGZO. Existing solutions reach their limits: interfacial doping layers or surface treatments are prone to damage during patterning and lack atomic-scale uniformity. This high R_C diminishes the intrinsic high mobility advantage of IGZO, making RC delays rather than carrier transit time the circuit speed limiter.

In this work, parasitic effects arising from the overlap regions within the device are inherently captured in TCAD simulations. For quantitative evaluation, specific parasitic parameters can be extracted via small-signal analysis. Inter-device parasitic capacitances in circuit-level simulations can be effectively represented as equivalent capacitance to ground, enabling comprehensive assessment of parasitic impacts. Moreover, self-heating and contact resistance effects can be explicitly incorporated in TCAD simulations, allowing for a unified analysis of their influence on device performance.

Note S11. Expansion of the monolithic 3D integration discussion

The following process flow illustrates the monolithic 3D (M3D) integration (**Figure S37**). In the proposed M3D integrated stack, the base layer is an advanced silicon CMOS with multiple Cu/low-k metal levels for routing. This is overlaid by two sequential TFT-based circuits layers (tier-1 and tier-2), each separated by low-k interlayer dielectrics (ILD, such as SiO_2) and connected by vertical vias. Each TFT consists of a bottom gate electrode (metal, deposited by sputter or e-beam evaporation), a high-k gate dielectric (such as HfO_2 by ALD), a semiconductor channel (such as IGZO and SnO, by sputter or ALD), and top source/drain (S/D) contacts (metal, by sputter or e-beam evaporation). All processing above the silicon substrate is restricted to < 400 °C to avoid damaging the CMOS metallization.

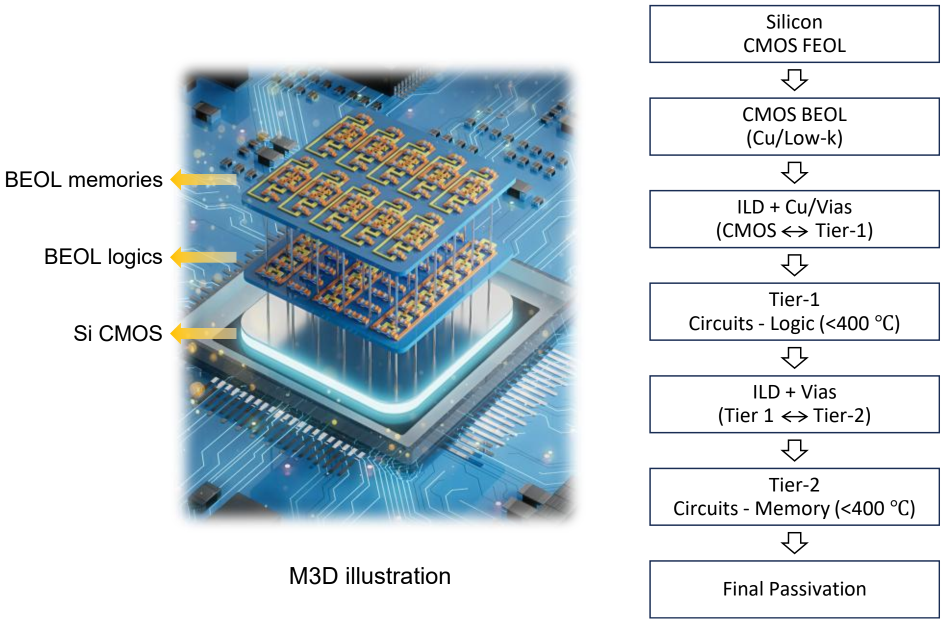

Figure S37. Proposed process flow diagram for M3D integration.

The n-channel devices (such as IGZO TFT) and p-channel devices (such as SnO TFT) are stacked into the tiers; circuit functionalities (CMOS logic, SRAM cell, etc.) are implemented using these TFTs. Each tier can thus support logic gates (inverters, NAND, etc.), memory elements (e.g. TFT-based SRAM or TFT + RRAM cells), and test circuits to evaluate performance. Importantly, the oxide-based TFT process is BEOL compatible because of its low thermal-budget deposition and no high-temperature dopant activation, which has been shown to yield high-performance TFTs without degrading the underlying silicon devices. By stacking such tiers and interconnecting them with dense vias, we can achieve a tightly integrated logic + memory system, thereby improving inter-layer bandwidth and enabling in-memory computing architectures. However, the M3D integration also faces some challenges.

Integration challenges:

Thermal budget: all upper layer processes are limited to 400 °C to protect BEOL metals. This low thermal budget can lead to lower material quality (e.g. less crystalline films) and accumulated stress in multilayers.

Thermal stress: repeated thermal cycles (even under 400 °C) and differing thermal expansion coefficients of the materials can lead to thermal stress. As each layer is heated and cooled, films may expand/contract differently, causing stress that could delaminate layers or crack the ILDs. Stacking multiple layers accumulates stress, and the top-level heating might induce stress at the via connections (thermal expansion mismatch between metal vias and surrounding dielectric).

Plasma damage: the plasma steps, including PEALD, RIE, and sputtering, can potentially damage sensitive thin films or underlying devices. High-energy ions or UV from the plasma may create defects in the IGZO or traps at dielectric interface, or induce charging that affects the CMOS below.

Via etch selectivity and alignment: etching the vertical inter-tier vias requires removing the ILD without damaging or mis-etching underlying layers. The etch must stop on a small metal pad often only tens of nanometer thick, without gouging into it or misaligning and hitting the surrounding devices. Selectivity of the etch chemistry between the ILD and the materials below (metals, oxides, etc.) is critical. Additionally, the vias are extremely small (nanometer scale), so alignment errors or etch undercut could cause opens or shorts.

Parasitic capacitance in stacked layers: stacking multiple device layers introduces parasitic capacitances between layers. For example, a tier-2 gate runs above a tier-1 source line, separated only by the ILD thickness, forming a capacitive coupling. Similarly, large metal planes (like ground references on different tiers) can overlay each other. These parasitic capacitances can slow down signals, increase power consumption, and cause signal crosstalk between tiers. In memory circuits (like 3D SRAM and RRAM), we must be mindful that vertical coupling cannot disturb stored data. The ILD dielectric constant and thickness heavily influence this coupling.

Device uniformity: ensuring each tier's transistors have similar characteristics is important (for logic threshold matching, SRAM stability, etc.). As tiers accumulate, slight process differences could cause threshold voltage or mobility variations between layers.

Thermal dissipation: stacked tiers could run hotter during operation because the top tiers are insulated by those below.

Note S12. The details of the TCAD device model calibration

The geometrical parameters of the device—such as structure, channel length/width, and thicknesses of the gate oxide and channel—were obtained based on experimental fabrication results. These parameters were fed into the Sentaurus Structure Editor tool for subsequent modeling. Other key material parameters were derived from literature and experimental measurements: the reference mobility values were selected based on experimental measurements and were fine-tuned during the calibration process; the electron affinity of IGZO were obtained from literature sources,[21] while the dielectric constant of gate oxide and bandgap of IGZO were derived from experimental results; relevant physical effects in IGZO were incorporated based on established studies.

The TCAD device model was rigorously calibrated across the subthreshold, linear, and saturation regimes to ensure accurate representation of device behavior over the entire operating ranges. In the subthreshold regime, D_{it} and generation-recombination parameters were adjusted to reproduce the experimentally measured SS and V_{TH}, with validation performed through quantitative comparison of simulated and measured data. In the linear regime, the low-field mobility model—incorporating both doping-dependent mobility and interface scattering effects—was iteratively refined, and the extracted transconductance was benchmarked against experimental values. In the saturation regime, high-field mobility saturation parameters were fine-tuned to match the experimentally observed saturation current, thereby further confirming model fidelity. Through this systematic multi-regime calibration, the TCAD framework was validated to reliably capture the device's electrical characteristics across its full operational spectrum.

References

[1] Y. Chen, D. Geng, T. Lin, M. Mativenga, J. Jang, *IEEE Electron Device Letters* **2016**, 37, 882.

[2] B. Tiwari, P. G. Bahubalindruni, A. Santos, A. Santa, C. Figueiredo, M. Pereira, R. Martins, E. Fortunato, P. Barquinha, *IEEE Journal of the Electron Devices Society* **2020**, 8, 584.

[3] J. R. Pradhan, M. Singh, S. Dasgupta, *Advanced Electronic Materials* **2022**, 8.

[4] T. Lei, R. Shi, Y. Wang, Z. Xia, M. Wong, *IEEE Transactions on Electron Devices* **2022**, 69, 3186.

[5] D. M. Sun, M. Y. Timmermans, A. Kaskela, A. G. Nasibulin, S. Kishimoto, T. Mizutani, E. I. Kauppinen, Y. Ohno, *Nat Commun* **2013**, 4, 2302.

[6] M. L. Geier, J. J. McMorrow, W. Xu, J. Zhu, C. H. Kim, T. J. Marks, M. C. Hersam, *Nat Nanotechnol* **2015**, 10, 944.

[7] J. A. Avila-Niño, E. R. Patchett, D. M. Taylor, H. E. Assender, S. G. Yeates, Z. Ding, J. J. Morrison, *Organic Electronics* **2016**, 31, 77.

[8] F. De Roose, K. Myny, M. Ameys, J.-L. P. J. van der Steen, J. Maas, J. de Riet, J. Genoe, W. Dehaene, *IEEE Journal of Solid-State Circuits* **2017**, 52, 3095.

[9] J. Yang, Y. Yuan, Y. Li, L. Du, Y. Wang, Z. Hu, Q. Wang, L. Zhou, Q. Xin, A. Song, *IEEE Electron Device Letters* **2018**, 39, 1876.

[10] W. Kim, S. Jung, *IEEE Electron Device Letters* **2022**, 43, 438.

[11] J. Zhang, W. Wang, J. Zhu, C. Wang, T. Zhu, C. Zhao, J. Wang, S. Zhang, X. Wang, K. C. Chang, H. Meng, M. Chan, M. Zhang, *ACS Nano* **2024**, 18, 3362.

[12] A. Ortiz-Conde, F. J. García-Sánchez, J. Muci, A. T. Barrios, J. J. Liou, C.-S. Ho, *Microelectronics Reliability* **2013**, 53, 90.

[13] P.-Y. Kuo, Z.-H. Li, C.-M. Chang, P.-T. Liu, *IEEE Transactions on Electron Devices* **2022**, 69, 4791.

[14] J. Guo, L. Wang, Y. Yu, P. Wang, Y. Huang, X. Duan, *Adv Mater* **2019**, 31, e1902962.

[15] X. Chen, S. Li, L. Zhu, J. Li, Y. Sun, N. Huo, *ACS Appl Mater Interfaces* **2024**, DOI: 10.1021/acsami.3c17572.

[16] J. Tauc, R. Grigorovici, A. Vancu, *physica status solidi (b)* **1966**, 15, 627.

[17] R. Engel-Herbert, Y. Hwang, S. Stemmer, *Journal of Applied Physics* **2010**, 108.

[18] S. M. Yoon, N. J. Seong, K. Choi, G. H. Seo, W. C. Shin, *ACS Appl Mater Interfaces* **2017**, 9, 22676.

[19] A. R. Choi, D. Kim, S. W. Ryu, S. Kim, I.-K. Oh, *Journal of Alloys and Compounds* **2025**, 182317.

[20] C. Wann, R. Wong, D. J. Frank, R. Mann, S.-B. Ko, P. Croce, D. Lea, D. Hoyniak, Y.-M. Lee, J. Toomey, presented at IEEE VLSI-TSA International Symposium on VLSI Technology, 2005.(VLSI-TSA-Tech). **2005**.

[21] G. Dastgeer, M. F. Khan, J. Cha, A. M. Afzal, K. H. Min, B. M. Ko, H. Liu, S. Hong, J. Eom, *Acs Appl Mater Inter* **2019**, 11, 10959.
